# NIR-Responsive Methotrexate-Modified Iron Selenide Nanorods for Synergistic Magnetic Hyperthermic, Photothermal, and Chemodynamic Therapy

**DOI:** 10.1021/acsami.3c18450

**Published:** 2024-05-13

**Authors:** Senthilkumar Thirumurugan, Kayalvizhi Samuvel Muthiah, Yu-Chien Lin, Udesh Dhawan, Wai-Ching Liu, An-Ni Wang, Xinke Liu, Michael Hsiao, Ching-Li Tseng, Ren-Jei Chung

**Affiliations:** † Department of Chemical Engineering and Biotechnology, National Taipei University of Technology (Taipei Tech), No. 1, Section 3, Zhongxiao East Road, Taipei 10608, Taiwan; ‡ Centre for the Cellular Microenvironment, Division of Biomedical Engineering, James Watt School of Engineering, Mazumdar-Shaw Advanced Research Centre, University of Glasgow, Glasgow G116EW, U.K.; § Faculty of Science and Technology, Technological and Higher Education Institute of Hong Kong, New Territories, Hong Kong 999077, China; ∥ Scrona AG, Grubenstrasse 9, 8045 Zürich, Switzerland; ⊥ College of Materials Science and Engineering, Chinese Engineering and Research Institute of Microelectronics, Shenzhen University, Shenzhen 518060, China; # Department of Electrical and Computer Engineering, National University of Singapore, Singapore 117583, Singapore; ∇ Genomics Research Center, Academia Sinica, Taipei 115, Taiwan; ○ Department and Graduate Institute of Veterinary Medicine, School of Veterinary Medicine, National Taiwan University, Taipei 10617, Taiwan; ◆ Graduate Institute of Biomedical Materials and Tissue Engineering, College of Biomedical Engineering, Taipei Medical University, Taipei 11031, Taiwan; ¶ International Ph.D. Program in Biomedical Engineering, College of Biomedical Engineering, Taipei Medical University, Taipei 11031, Taiwan; ⋈ Research Center of Biomedical Device, College of Biomedical Engineering, Taipei Medical University, Taipei 11031, Taiwan; ⧓ International Ph.D. Program in Cell Therapy and Regenerative Medicine, College of Medicine, Taipei Medical University, Taipei 11031, Taiwan; ⧖ High-Value Biomaterials Research and Commercialization Center, National Taipei University of Technology (Taipei Tech), Taipei 10608, Taiwan

**Keywords:** magnetic hyperthermia therapy (MHT), photothermal therapy (PTT), alternating magnetic field (AMF), chemodynamic therapy (CDT), methotrexate (MTX)

## Abstract

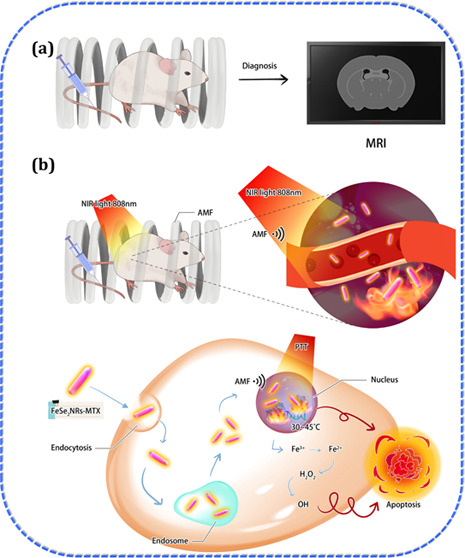

Breast cancer is a malignant tumor with a high mortality rate among women. Therefore, it is necessary to develop novel therapies to effectively treat this disease. In this study, iron selenide nanorods (FeSe_2_ NRs) were designed for use in magnetic hyperthermic, photothermal, and chemodynamic therapy (MHT/PTT/CDT) for breast cancer. To illustrate their efficacy, FeSe_2_ NRs were modified with the chemotherapeutic agent methotrexate (MTX). MTX-modified FeSe_2_ (FeSe_2_-MTX) exhibited excellent controlled drug release properties. Fe^2+^ released from FeSe_2_ NRs induced the release of ^•^OH from H_2_O_2_ via a Fenton/Fenton-like reaction, enhancing the efficacy of CDT. Under alternating magnetic field (AMF) stimulation and 808 nm laser irradiation, FeSe_2_-MTX exerted potent hyperthermic and photothermal effects by suppressing tumor growth in a breast cancer nude mouse model. In addition, FeSe_2_ NRs can be used for magnetic resonance imaging in vivo by incorporating their superparamagnetic characteristics into a single nanomaterial. Overall, we presented a novel technique for the precise delivery of functional nanosystems to tumors that can enhance the efficacy of breast cancer treatment.

## 1 Introduction

Breast cancer is a lethal disease in women that spreads to other organs, including the lungs and bones. Breast cancer affects approximately one in every eight women during their lifetime,^1,2^ and surgery,^3^ radiation treatment,^4,5^ and chemotherapy are the primary treatment options for the disease. However, these treatment methods have limitations, including surgical risks and the substantial side effects of radiotherapy and chemotherapy.^6^ Additionally, these treatment strategies result in a high rate of local recurrence or distant metastasis, making complete tumor resection difficult and decreasing patient survival. Therefore, novel therapeutic strategies for breast cancer should be developed.^7^

Recently, chemotherapy has been combined with various therapeutic modalities, such as magnetic hyperthermia therapy (MHT), photothermal therapy (PTT), gene therapy, and radiotherapy, to achieve synergistic cancer therapy.^8,9^ MHT is based on the induction heating of magnetic nanoparticles (MNPs) in the presence of an external magnetic field. MNPs are crucial for various biomedical applications, such as drug delivery, MHT, and magnetic resonance imaging (MRI).^10,11^ MHT has been established as a cancer treatment technique over the past decade by maintaining the temperature of tumor cells at 41–46 °C to block the regulatory and growth activities of cancer cells.^12,13^ MNPs are frequently incorporated into polymer systems, such as hydrogel liposomes and micelles, to magnetically guide the nanoparticles to the tumor site^14,15^ or facilitate controlled drug release in a magnetic field under hyperthermia by an alternating magnetic field (AMF).^16^

PTT is a highly successful cancer treatment strategy that transforms near-infrared (NIR) light energy into heat using a photothermal agent, thereby increasing the temperature at the tumor site.^17^ Cancerous cells are more susceptible to PTT than healthy cells.^18,19^ In addition, PTT can indirectly boost the efficacy of other therapeutic techniques because localized heat increases the permeability of both the tumor vasculature and tumor cell membranes, facilitating nanoparticle accumulation and uptake by the targeted tissue/cell. PTT has several advantages, including excellent spatiotemporal resolution, safety, efficacy, and noninvasive therapy.^20,21^ Various nanomaterials, including metallic nanostructured materials (Au, Ag, and Pd), carbon-based nanostructured materials (carbon spheres, nanotubes, and graphene oxide), transition-metal dichalcogenide nanostructured materials (WS_2_, MoS_2_, and WSe_2_), metal oxide nanoparticles (WO_3_x and MoO_3_x), and polymer nanoparticles (polypyrrole),^22,23^ exhibit substantial localized surface plasmon resonance absorption in the NIR region, with large metallic nanostructures and highly self-doped semiconductors or have a suitably low band gap. However, not all nanomaterials are suitable for clinical application.^24^ CDT is a cancer therapeutic approach based on Fenton or Fenton-like reactions that convert physiological hydrogen peroxide (H_2_O_2_) into a highly lethal hydroxyl radical (^•^OH).

Fe and Se are used in cancer treatment because of their biocompatibility.^25^ Selenium, a trace element, has recently garnered attention because of its numerous health benefits, particularly those related to immune function and cancer prevention.^26,27^ However, the concentration required to function as an antitumor agent and important microelement is close to hazardous levels in selenium, which severely limits its therapeutic use.^28,29^ Despite this, selenium nanoparticles (nano-Se) are gaining attention owing to their high bioavailability, biological activity, and low toxicity.^30,31^ The toxicity of elemental selenium (Se^0^) nanoparticles is lower than that of selenite (Se^2+^ or Se^4+^) ions. Therefore, selenium nanoparticles may replace other forms of selenium in dietary supplements and pharmacological drugs.^32^ Iron-containing nanoparticles are widely used for MRI,^33^ and FeSe_2_, a crucial group of transition-metal dichalcogenides, has attracted considerable interest owing to its superior magnetic characteristics, electrical conductivity, and NIR absorption.^34^ FeSe_2_ comprises Fe^2+^ ions, which oxidize quickly into Fe^3+^ ions, both in vivo and in vitro.^35^ Compared to inert virus-like silica nanoparticles, strong catalytic properties, photoresponsiveness, and low toxicity make FeSe_2_ nanoparticles excellent candidate therapeutic agents. Additionally, compared to other known magnetic materials, such as Fe_2_O_3_, Fe_3_O_4_, and MFe_2_O_4_ (where M = Mn or Co) nanoparticles, FeSe_2_ nanoparticles exhibit unique functional properties, such as a broad absorption spectrum spanning the NIR-II^36^ range and high morphological tunability, implying better suitability for biomedical and therapeutic engineering. Therefore, the combination of chemotherapy and MHT/PTT/CDT is expected to exert synergistic therapeutic effects without harming healthy tissues. To achieve such synergistic effects, it is essential to develop multifunctional systems capable of controlling the drug delivery and MHT/PTT effects.^37^

Improving the therapeutic index of a drug by increasing its selective uptake into target cells is the primary goal of targeted drug delivery.^38^ Methotrexate (MTX), a folic acid analogue, impairs cellular folate metabolism by blocking dihydrofolate reductase. MTX is a potential candidate for the treatment of tumors with excessive folate receptor expression on their surface. After crossing the cell membrane, MTX undergoes rapid intracellular bioconversion to a polyglutamate derivative via folyl polyglutamyl synthase. This bioactivation considerably increases the pharmacological action of MTX by prolonging its intracellular retention and increasing its inhibitory activity, thereby inhibiting the cellular synthesis of DNA and RNA building blocks and inducing cell apoptosis.^39^ MTX is used in dual-role compounds that can function as both tumor-targeted binding sites and therapeutic drugs.

**Scheme 1:**
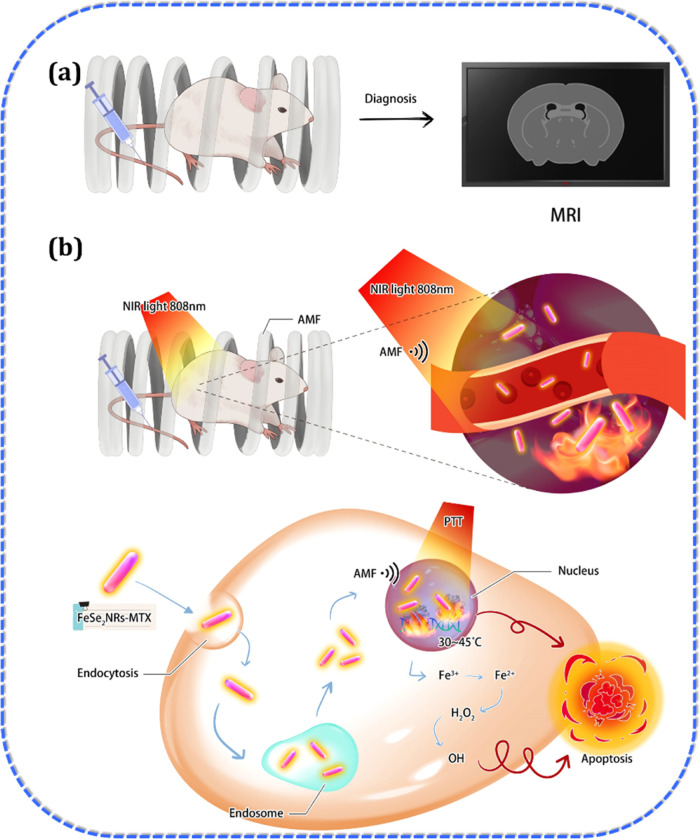
Schematic Illustrations of FeSe_2_-MTX Applications in MRI Combined with Magnetic Hyperthermic, Photothermal, and Chemodynamic Therapy (MHT/PTT/CDT)

In this study, we found that the heating efficiency of FeSe_2_ nanorods (NRs) was maximized by the simultaneous application of AMF, CDT, and NIR laser irradiation. Dual-heating effects were investigated in aqueous suspensions, in vitro tumor cells, and in vivo solid tumors. All of the tested treatments exerted cumulative or synergistic effects. NRs exert potent heating effects on suspensions and cancer cells in vitro. Although each type of heat alone caused only a moderate amount of cytotoxicity and killed some cancer cells, their combination (MHT/PTT/CDT) killed all cancer cells. Therefore, the dual-heating method eliminated solid tumors in the mice. As illustrated in Scheme 1, FeSe_2_-MTX efficiently enters cancer cells via endocytosis. Acidification and perturbation result in the destabilization of biological membranes, leading to the endosomal escape of FeSe_2_-MTX. Furthermore, FeSe_2_-MTX leaves the cytoplasm and enters the nucleus, and MHT/PTT/CDT synergistically induces apoptosis, thereby enhancing anticancer efficacy.

## 2 Materials and Methods

### 2.1 Preparation of FeSe_2_ NRs

Briefly, a 40 mL aqueous solution was prepared by dissolving (Fe[NO_3_])_3_·9H_2_O (75 mM), Na_2_SeO_3_ (75 mM), and hydrazine hydrate (N_2_H_4_·H_2_O; 1.5 M) in Milli-Q water with continuous stirring. These solutions were then placed in a 50 mL stainless-steel Teflon-lined autoclave and incubated at 140 °C for 12 h. After naturally cooling to ambient temperature, black precipitates were filtered out, washed multiple times with distilled water, mixed with 100% ethanol, and dried under vacuum at 50 °C for 6 h.

### 2.2 Drug Release

The release of MTX from the MTX-loaded FeSe_2_ NRs was examined in an aqueous environment. Briefly, 20 mg of drug-loaded NRs was dissolved in 10 mL of phosphate-buffered saline (PBS), transferred to a dialysis membrane (molecular weight (MW): 12 000 Da), and incubated with 100 mL of PBS (pH 7.4) solution in a shaker at 50 rpm and 37 °C for 24 h. At specified intervals (0.5, 1, 2, 3, 6, 12, 24, 48, and 72 h), the suspension (3 mL) was removed from the aliquots and replaced with an equivalent volume of freshly prepared PBS. The MTX concentration was determined by spectrophotometry at a maximum wavelength of 305 nm. Next, the release of MTX from MTX-loaded FeSe_2_ NRs was assessed in a slightly acidic environment (pH 6.4).

### 2.3 Temperature Elevation

To determine the ability of FeSe_2_ NRs to produce heat in response to magnetic field stimulation, varying concentrations of FeSe_2_ NPs (0.625, 1.25, 2.5, 5.0, and 10 mg/mL) were dissolved in distilled water, placed in a 1.5 mL Eppendorf, and exposed to AMF (700–1100 kHz) for 10 min. The temperatures of all samples were recorded every 30 s.

### 2.4 Photothermal Evaluation of NRs

The photothermal effects of FeSe_2_ NRs in PBS were determined using a fiber-coupled diode laser (808 nm). Images were captured using an infrared thermal imaging system during laser irradiation. The photothermal effects of FeSe_2_ NRs at different concentrations (0, 0.125, 0.25, 0.5, 1, and 2 mg/mL) were also determined.

### 2.5 In Vitro Synergistic Combination Therapy

MCF-7 cells were seeded at a density of 5 × 10^4^ cells in a 35 mm dish. After 12 h of incubation, cells were attached to the bottom of the dish and washed twice with PBS, and the FeSe_2_-MTX suspension (100 μg/mL in Dulbecco’s modified Eagle’s medium (DMEM)) was added to the 35 mm dish and incubated for another 4 h for internal uptake of nanoparticles. Cells were divided into different groups: (i) control, (ii) AMF, (iii) 808 nm laser, and (iv) AMF + laser + CDT. They were treated for 5 min and incubated for 8 h. Next, 100 μL of 3-(4,5-dimethylthiazol-2-yl)-2,5-diphenyltetrazolium bromide (MTT) solution was added, and the cells were incubated for another 2 h to determine the cell viability.

### 2.6 Live/Dead Assay

Briefly, 1 × 10^5^ MCF-7 cells were seeded into a 12-well plate and cultured for 24 h. The MCF-7 cells were incubated with the prepared materials for 4 h. Subsequently, the cells were exposed to specific treatments: 5 min of irradiation with an 808 nm laser (output maximum energy 2.0 W/cm^2^ with a fixed and proper distance), 5 min of AMF treatment, and MHT/PTT/CDT treatment (5 min each). After another 12 h of incubation, the cells were stained using a live/dead cell staining kit according to the manufacturer’s protocol and visualized under an inverted fluorescence microscope (FM, KA0901; Abnova, Taiwan).

### 2.7 Animals

Healthy BALB/c nude mice (4–6 weeks old) were acquired from BioLasco (Taipei, Taiwan). Tumors were induced by subcutaneously injecting a suspension of 2 × 10^6^ MCF-7 cells into mice. All experiments were conducted with the approval of the Institutional Animal Care and Use Committee of Taipei Medical University (LAC2022-0445).

### 2.8 Imaging of Nanoparticles

MRI was performed using a 7.0 T MRI scanner. First, gradient concentrations of FeSe_2_-MTX were introduced into the tube, and their contrast values were determined. FeSe_2_-MTX dispersions were then intravenously injected into nude mice for in vivo MRI evaluation. MRI images of the mice before and after injection of the materials were acquired and evaluated.

### 2.9 In Vivo MHT/PTT Evaluation

Mice were randomly divided into five groups (*n* = 6/group) and treated with (1) PBS, (2) FeSe_2_-MTX, (3) FeSe_2_-MTX plus AMF, (4) FeSe_2_-MTX + PTT, and (5) FeSe_2_-MTX plus MHT/PTT/CDT. Magnetic hyperthermia experiments were performed using an inductive coil. During the hyperthermia treatment, the animals were anesthetized and placed inside the coil with an ac magnetic field (frequency of 700–1100 kHz) and/or laser irradiation of 808 nm at 0.75 W/cm^2^ for 5 min. Notably, 100 μL of FeSe_2_-MTX was delivered intravenously into the mice. During therapy, the prepared nanoparticles were injected and exposed to various treatments. The tumor volume and body weight were recorded. After the 21-day treatment period, the mice were sacrificed for subsequent analysis.

### 2.10 Histological Analysis

After treatment, the heart, liver, spleen, lungs, and kidneys were removed, stained with hematoxylin and eosin (H&E), and observed under a microscope.

## 3 Results and Discussion

### 3.1 Characterization of NRs

**Figure 1:**
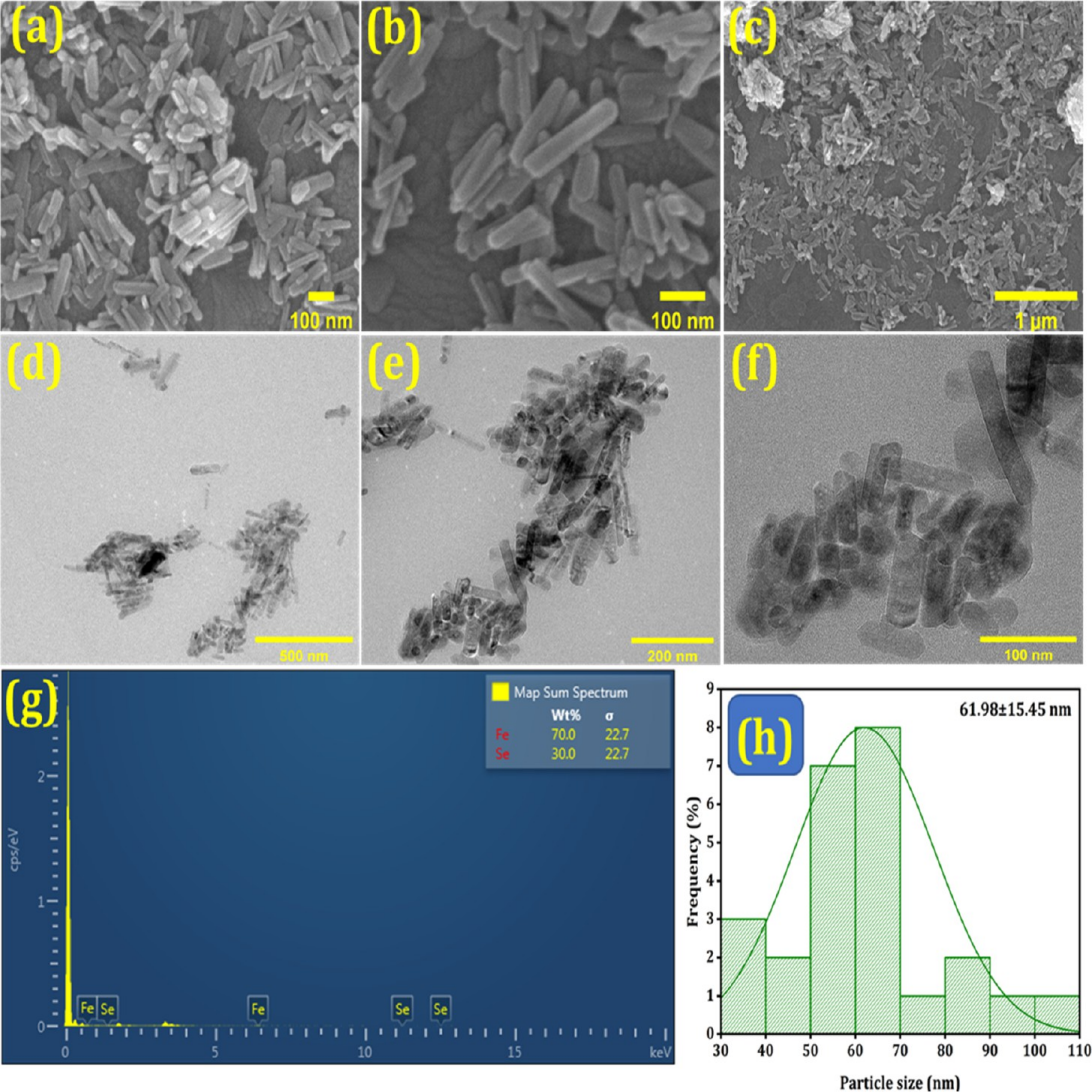
Characterization of nanorods (NRs). (a–c) FE-SEM images of FeSe_2_ NRs. (d–f) TEM images of FeSe_2_ NRs. (g) EDX analysis of FeSe_2_ NRs. (h) Particle size distribution curve of NRs.

**Scheme 2:**
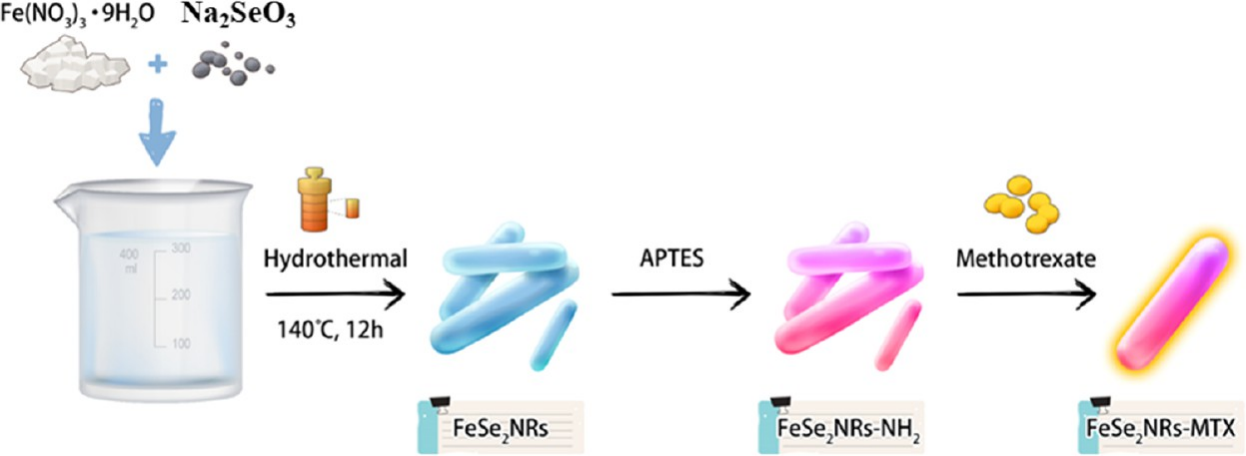
Schematic Illustration of the Hydrothermal Synthesis of FeSe_2_-MTX

Magnetic FeSe_2_ NRs were hydrothermally prepared via a single reaction. As depicted in Scheme 2, sodium selenide and Fe(NO_3_)_3_·9H_2_O were combined with 60 mL of DI water and allowed to dissolve. Subsequently, hydrazine solution was added to the mixture while stirring magnetically at ambient temperature. Subsequently, this solution was transferred to a Teflon-lined autoclave and heated at 140 °C for 12 h to produce FeSe_2_ NRs. FeSe_2_-MTX was then formed by adding MTX to the FeSe_2_-NR solution. Field emission scanning electron microscopy (FE-SEM) and transmission electron microscopy (TEM) were used to determine the surface morphology and size of the resulting NRs. High-magnification FE-SEM (Figure 1a–c) and TEM (Figure 1d–f) images of the nanocarrier platform (FeSe_2_) revealed the rod-shaped morphology of the nanoparticles. This image shows that the diameter of the particles was in the nanometer range. Figure 1g shows the energy-dispersive X-ray (EDX) analysis of the FeSe_2_ NRs containing elemental weight percentages of 70% Fe and 30% Se. Figure 1h depicts the rod-shaped particles with a diameter of 61.98 ± 15.45 nm. The hydrodynamic size distribution was analyzed by dynamic light scattering (DLS) (Figure S4). As shown in Figure S4, the average particle size (diameter) of the FeSe_2_ NRs was 91 nm, and the polydispersity index (PDI) was 0.28. The PDI was below 0.30, providing evidence of a uniform particle size distribution in addition to the good suspension stability of the NPs. For drug delivery, a PDI value of 0.3 or below was considered acceptable, which indicates a homogeneous distribution.^40^ Nanoparticles require more efficient cell penetration, whereas larger nanoparticles require additional energy and driving forces for cellular internalization.^41^ Nanoparticle shape influences cellular uptake. For example, nanorods are more efficiently internalized into epithelial cells than spheres.^42,43^ Cancer cells engulf nanorods in a vesicle through an endocytic process called macropinocytosis.^44^ It can internalize the relatively large nanosized particles with diameters of up to 5 μm.^45,46^ To evaluate the colloidal stability of NPs, we determined their ζ potential (Figure S5). They were found to be −21.5 and −18.9 mV for FeSe_2_ and FeSe_2_-MTX, respectively. The ζ potential was determined using a Zetasizer or other means and provides information on the charge of the particles and the tendency of the particles in a formulation to aggregate or remain discrete. Particles with ζ potentials of more than −30 mV are considered stable.^47^ The NPs were stable for the duration of the 1-week study. However, a negative ζ potential is preferable to promote the enhanced retention and permeability of nanoparticles by evading recognition by macrophages and reducing protein adsorption on the surface of the nanoparticle, which elicit an immune response and subsequent immune-mediated deleterious side effects. Thus, we confirmed that the NPs were stable under physiological conditions.

**Figure 2:**
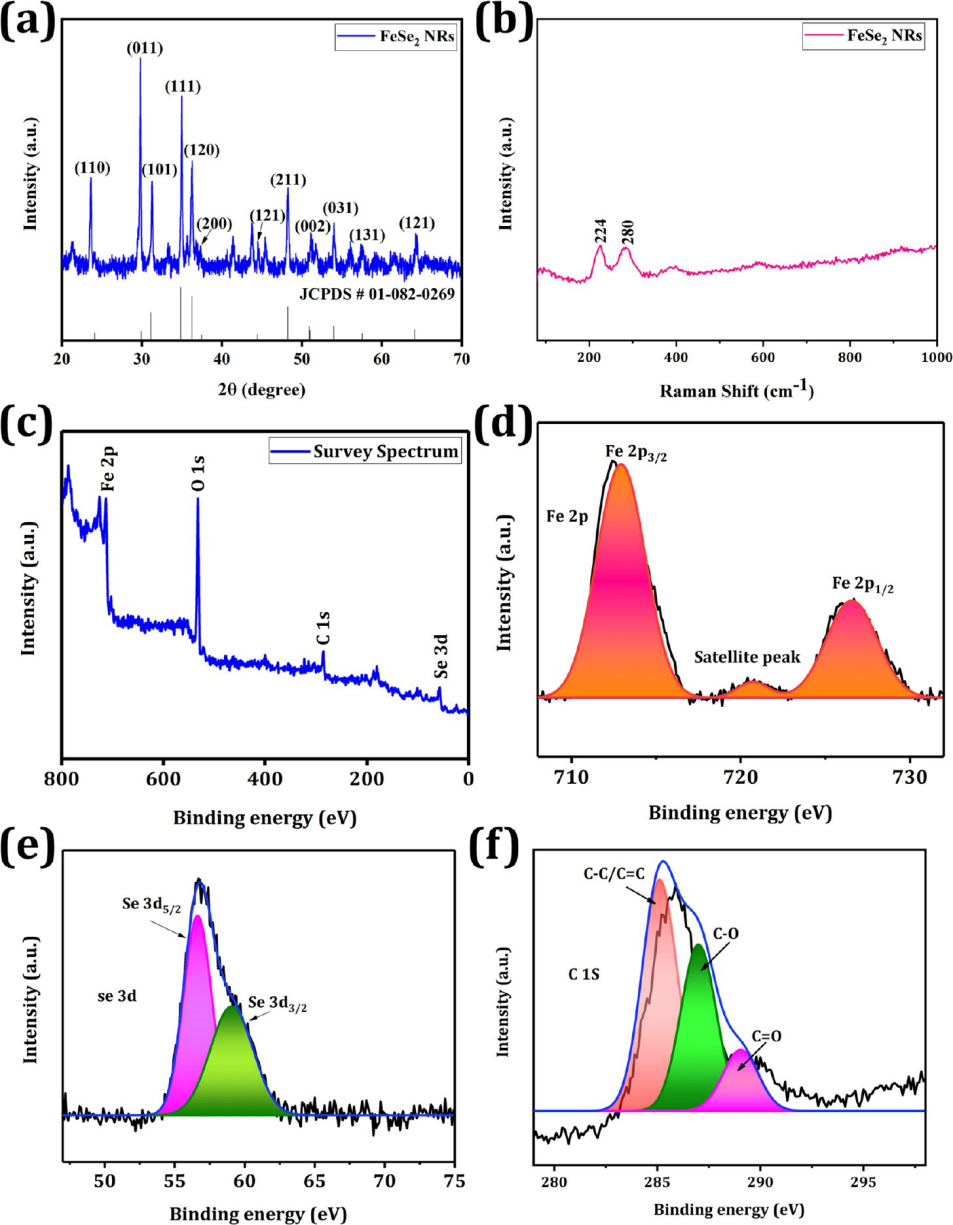
Characterization of NRs. (a) XRD spectra of FeSe_2_ NRs. (b) Raman spectra of NRs. (c) Survey spectra of NRs. (d) High-resolution Fe 2p spectra. (e) High-resolution Se 3d spectra of NRs. (f) High-resolution C 1s spectra of NRs.

The crystallinity of the FeSe_2_ NRs was further studied using X-ray diffraction (XRD), and the resulting patterns matched the orthorhombic crystal structure of FeSe_2_ (JCPDS no. 01-082-0269), as shown in Figure 2a. Fourier-transform infrared (FTIR) spectroscopy was used to determine the interactions between MTX and FeSe_2_ NRs (Figure S3). The peak at 800–870 cm^–1^ was associated with the C–O–C group of FeSe_2_ NRs. After anime modifications, the peaks at 1649 and 1628 cm^–1^ corresponded to the −NH vibration of 3-aminopropyltriethoxysilane (APTES).^48^ Several distinctive vibration absorptions were identified in the free MTX spectra (Figure S1). The peak at 3394 cm^–1^ suggested the presence of an NH group. The presence of carboxylic acid was indicated by absorption peaks at 3060 and 2951 cm^–1^. The peaks between 1500 and 1700 cm^–1^ correspond to C–N or NH_2_ vibrations, whereas the peaks at 1490 cm^–1^ indicate C–C bond stretching vibrations.^49^ The FTIR spectrum of FeSe_2_-MTX showed a small number of distinctive peaks, indicating that MTX was successfully modified onto the FeSe_2_ NRs.

Figure 2b depicts the results of the Raman spectroscopy studies of the FeSe_2_ NRs using a 632.8 nm laser source. Raman active modes of FeSe_2_ NRs were distinct at 224 and 280 cm^–1^. Lutz and Muller obtained Raman spectra for FeSe_2_ marcasite, assigning peaks at 221 and 264 cm^–1^ to the A_g_ and B_1g_ Se–Se stretching modes, respectively. The vibrational peaks of the Se–Se atoms are very similar to those previously reported for nanocrystalline FeSe_2_.^50^ The MTX-conjugated NPs were analyzed using UV–visible spectroscopy to confirm that MTX was successfully conjugated to the nanorods. Figure S4 shows the UV–visible spectra of MTX-conjugated NRs and an aqueous solution of free MTX. MTX-conjugated NRs showed a characteristic absorbance peak similar to that of free MTX, confirming the presence of MTX on the NR surface.

The surface elemental composition and oxidation states of the FeSe_2_ NRs were investigated using X-ray photoelectron spectroscopy (XPS) in the range 0–800 eV. The XPS survey revealed distinct Fe 2p, Se 3d, C 1s, and O 1s spectra (Figure 2c). The high-resolution Fe 2p spectrum was deconvoluted into three peaks at 713.2, 720.5, and 726.6 eV, which were equivalent to the Fe^2+^ oxidation state in Fe 2p_3/2_, the satellite peak, and Fe 2p_1/2_, respectively (Figure 2d).^51^ High-resolution Se 3d spectrum was divided into two peaks at 55.6 and 58.8°, which were attributed to Se 3d_5/2_ and Se 3d_3/2_, respectively, indicating the −2 valence state of Se (Figure 2e).^52^ In addition, high-resolution C 1s spectra were deconvoluted into three peaks at 284.6, 286.4, and 288.6 eV, which were consistent with the C–C/C=C, C– O, and C=O bonds, respectively (Figure 2f).^53^ O 1s spectra in Figure S5 were split into two peaks at 534.2 and 532.3 eV, corresponding to the epoxy C–O groups in graphene and Se–O, respectively.^54,55^ Therefore, XPS results confirmed the successful synthesis of FeSe_2_ NRs.

### 3.2 Magnetic Analysis and CDT Effects of NRs

#### 3.2.1 Magnetic Analysis of NRs

**Figure 3:**
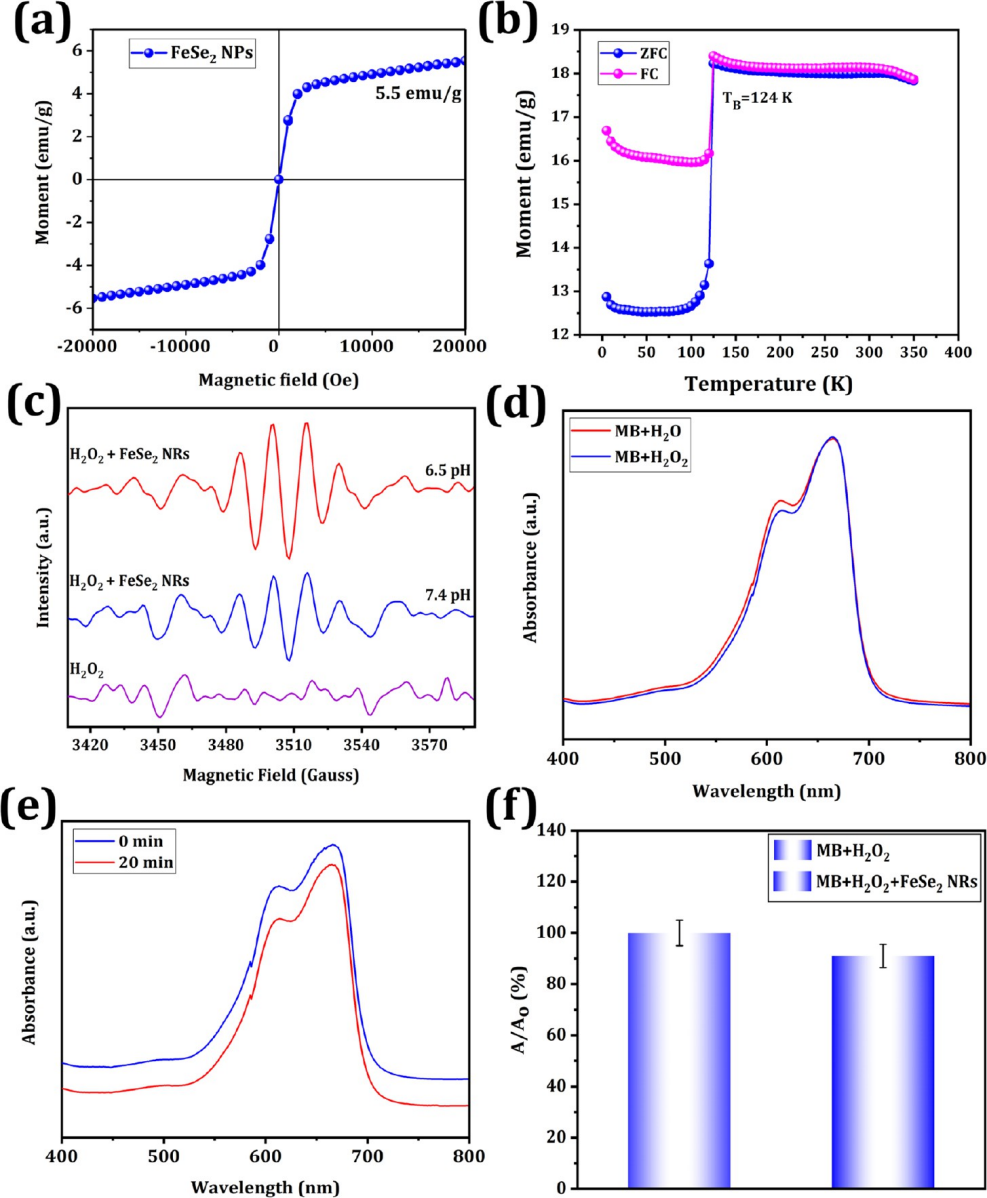
Magnetic analysis of NRs. (a) *M*–*H* curve of FeSe_2_ NRs. (b) *M*–*T* curve of FeSe_2_ NRs. CDT analysis using the UV–visible spectra of methylene blue (MB) solution after treatment with (c) H_2_O_2_ and (d) NRs + H_2_O_2_. (e) Histogram analysis of MB degradation. (f) Electron spin resonance (ESR) analysis.

Figure 3a depicts the hysteresis curve between the applied magnetic field and the magnetization (*M*–*H*) curve at ambient temperature. The as-prepared NRs exhibit an S-shaped curve, confirming their superparamagnetic properties. Superparamagnetic materials exhibit zero coercivity and retention. At room temperature, the saturation magnetization (*M*_s_) of the prepared NRs was 5.5 emu/g. *M*_s_ increased as the temperature increased and then decreased as the temperature increased (Figure 3a), suggesting that the magnetization diminished when the crystallite size and temperature increased. Therefore, superparamagnetic nanoparticles can be utilized for in vivo applications as they do not maintain magnetization before and after exposure to the applied field and have a lower chance of aggregating.^56^ The zero-field-cooling (ZFC) and field-cooling (FC) curves of the prepared FeSe_2_ NRs are shown in Figure 3b. The ZFC–FC curve illustrates the behavior of the magnetic moment spin fluctuations at various temperatures. These curves were used to calculate blocking temperatures (TB). The TB of the prepared FeSe_2_ NRs was 124 K. These NRs exhibited ferromagnetism below TB and superparamagnetism beyond TB.^57^

#### 3.2.2 CDT Effects of NRs

An ^•^OH signal was detected via electron spin resonance (ESR) spectroscopy using 5,5-dimethyl-1-pyrroline *N*-oxide (DMPO) as a trapping agent, which showed four peaks with an intensity of approximately 1:2:2:1. As depicted in Figure 3c, there was no discernible variation in the characteristic peaks of ^•^OH in the H_2_O_2_ solution, but the characteristic peaks appeared after the addition of the substrate H_2_O_2_ to the FeSe_2_ NRs, indicating that the FeSe_2_ NRs reacted with H_2_O_2_ at neutral pH (pH 7.4) to generate less ^•^OH. There was a considerable increase in the ESR intensity at pH 6.5, suggesting that a mildly acidic environment increased the production of ^•^OH. To examine the activity of FeSe_2_ NRs in converting H_2_O_2_ to ^•^OH in an acidic environment, we determined ^•^OH through MB degradation. According to the UV–vis spectra, H_2_O_2_ had no effect on the absorption of MB in an acidic environment, as shown in Figure 3d. When MB was incubated with H_2_O_2_ and FeSe_2_ NRs for 20 min at pH 6.5, a decreased absorbance peak for MB was recorded, which could be attributed to ^•^OH formation via a Fenton-like reaction. A decrease in the maximum absorbance peak of MB is shown in Figure 3e. These findings demonstrate that FeSe_2_ NRs can catalyze the conversion of H_2_O_2_ to ^•^OH in an acidic environment, confirming that they are potential CDT agents to kill cancer cells. In addition, the maximum absorption of MB decreased from 100 to 91% after incubation with H_2_O_2_ only and H_2_O_2_ and FeSe_2_ NRs (Figure 3f). These results indicate the efficacy of the FeSe_2_–NR-based Fenton-like reaction in increasing ^•^OH production, which can be used for further in vitro and in vivo development of CDT.

### 3.3 Hyperthermia Analysis

#### 3.3.1 Photothermal Effects of NRs

**Figure 4:**
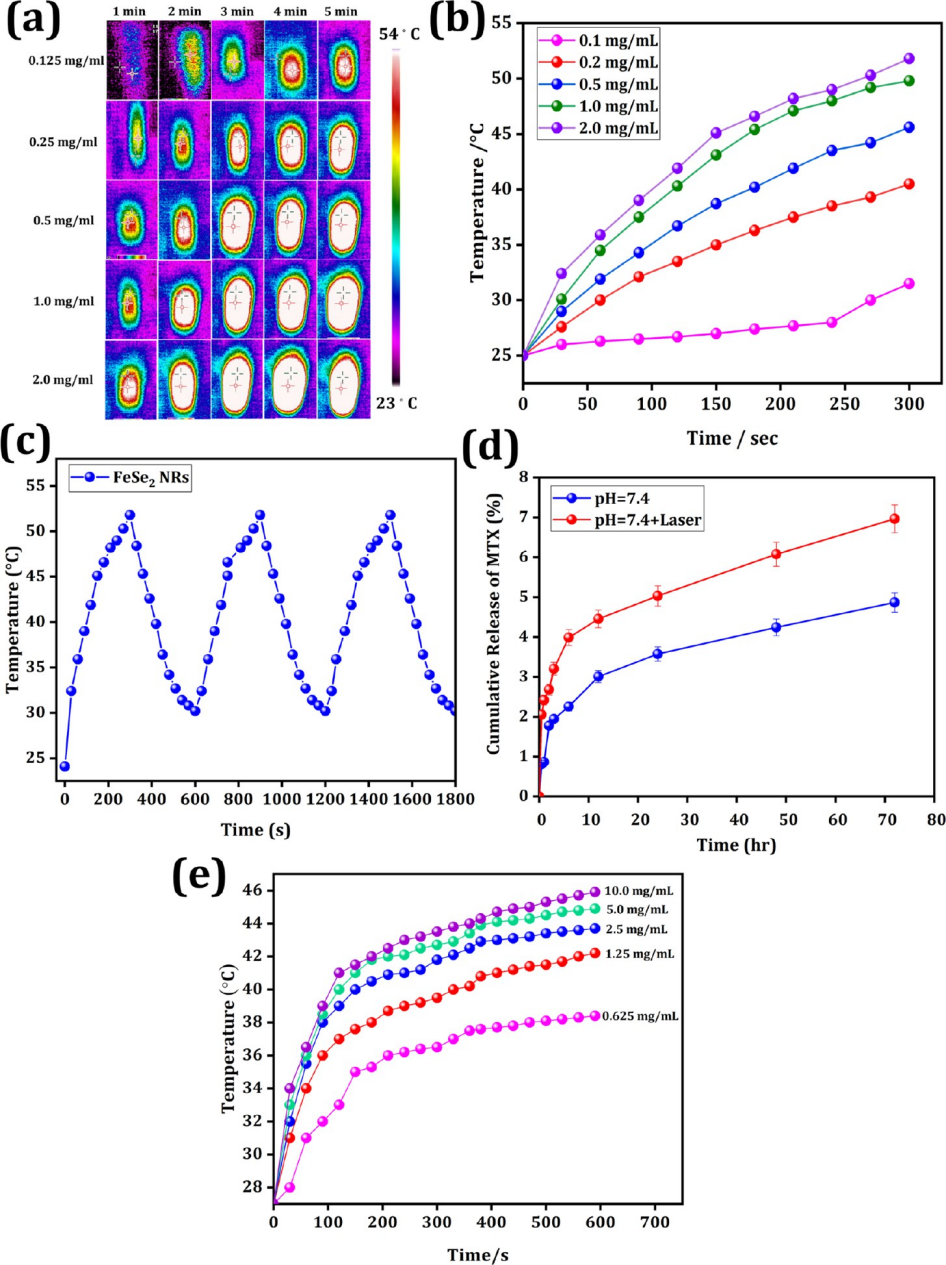
Photothermal effects of FeSe_2_ NRs. (a) IR images of NRs at different concentrations and time intervals under NIR. (b) Temperature elevation curves of NRs at different concentrations under NIR (2 W/cm^2^). (c) On and off cycle temperature profiles of NRs. (d) In vitro drug release profiles of FeSe_2_-MTX with and without NIR. (e) Heat-generating capacity of FeSe_2_ NRs on exposure to AMF; 3.2 kW, 700–1100 kHz.

An infrared (IR) thermal camera was used to study photothermal performance and conversion efficiency. After 5 min of 808 nm laser irradiation, the IR photographs in Figure 4a indicate that the FeSe_2_ NRs displayed superior photothermal performance and conversion capability, as a large temperature gradient could be observed. We determined that the PTT efficiency of the FeSe_2_ NRs was concentration- and laser power-dependent by comparing the PTT heating profiles over time, as shown in Figure 4b. After 5 min of 808 nm laser irradiation with a power density of 2.0 W/cm^2^ (output energy) and a concentration of 2 mg/mL, the temperature of FeSe_2_ NR dispersions increased by approximately 52 °C. The synthesized NRs exhibited excellent thermal stability over three heating and cooling cycles, as shown in Figure 4c. The photothermal conversion efficiency of FeSe_2_ NRs was 35.5%. Therefore, the results show that these FeSe_2_ NRs are promising candidates for application as theranostic agents in cancer therapy.

To test the drug release behavior, the anticancer agent MTX was loaded onto the FeSe_2_ NRs as a model drug. Figure 4d demonstrates that the cumulative MTX release from FeSe_2_-MTX in a solution of pH 7.4 was 4%, whereas 7% more MTX molecules were released in the presence of the NIR laser. Under pH 6.4 (Figure S6), drug release was higher than that at pH 7.4, mimicking the pH states of blood circulation and the tumor microenvironment. This was because MTX, an anticancer drug, was covalently functionalized onto the nanoparticles through APTES grafting and the effect of hyperthermia on the dissociation of methotrexate from the FeSe_2_-MTX conjugate.^58^ These results suggest that NIR irradiation improves the efficiency of drug delivery to malignant tissues.

#### 3.3.2 Heat-Generating Capability of NRs

The heat-generating properties of FeSe_2_ NRs were evaluated using MHT and laser irradiation (808 nm). The effect of MHT on FeSe_2_ NRs was investigated. AMF was applied to a series of NR solutions at concentrations of 0.625, 1.25, 2.5, 5.0, and 10 mg/mL for 5 min (3.2 kW, 700–1100 kHz). As shown in Figure 4e, the magnetic hysteresis loss and the Neel and Brownian relaxations of every nanoparticle activated by an external AMF resulted in accelerated temperature expansion.^59^ Superparamagnetic suspensions generate heat energy for cancer treatment with high efficiency.^60^ Although cancer cells are much more temperature-sensitive than healthy cells, they are easily eliminated at the ideal temperature. Overall, these findings suggest that the synthesized NRs are effective agents for treatments using MHT.

### 3.4 Cellular Uptake

**Figure 5:**
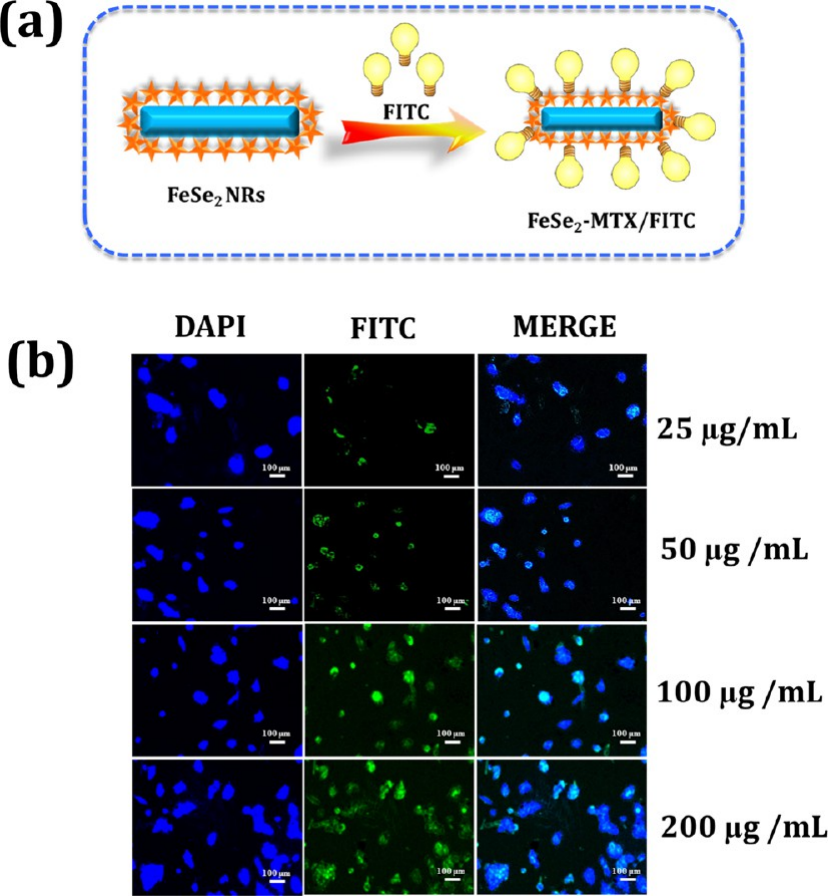
(a) Scheme for FITC conjugation with FeSe_2_-MTX. (b) Dual-labeling FM images of MCF-7 cancer cells after treatment with varying concentrations of FeSe_2_-MTX (25, 50, 100, and 200 μg/mL). Green fluorescence indicates FeSe_2_-MTX/FITC accumulation. Blue fluorescence indicates DAPI-stained nuclei.

A schematic diagram of the conjugation of fluorescein isothiocyanate (FITC) to FeSe_2_-MTX is shown in Figure 5a. Labeled cells were incubated with FeSe_2_-MTX/FITC at different concentrations of 25, 50, 100, and 200 μg/mL for 12 h. The accumulation of FeSe_2_-MTX/FITC and 4′,6-diamidino-2-phenylindole (DAPI) nuclear staining revealed green and blue fluorescence, as presented in Figure 5b. In addition, the ingestion of FeSe_2_-MTX/FITC by MCF-7 cells significantly increased when the nanocarrier system concentration was increased, indicating that cellular uptake was concentration-dependent. As shown in Figure 5b, the DAPI and FITC signals confirmed the uptake of FeSe_2_-MTX/FITC by MCF-7 cells during incubation. These findings suggest that nanoparticles successfully permeate the nucleus of a cell, where MTX binds to DNA, owing to the higher affinity of MTX-conjugated nanoparticles for folic acid receptors expressed on the surface of cancer cells.^61^ As mentioned, the physicochemical characteristics of the nanoparticles play a crucial role in the uptake process. These findings indicated that the nanosystem was sufficiently stable to bypass the endosome and disseminate in the nuclei after drug release.

### 3.5 In Vitro MHT/PTT/CDT Synergistic Therapeutic Effect

**Figure 6:**
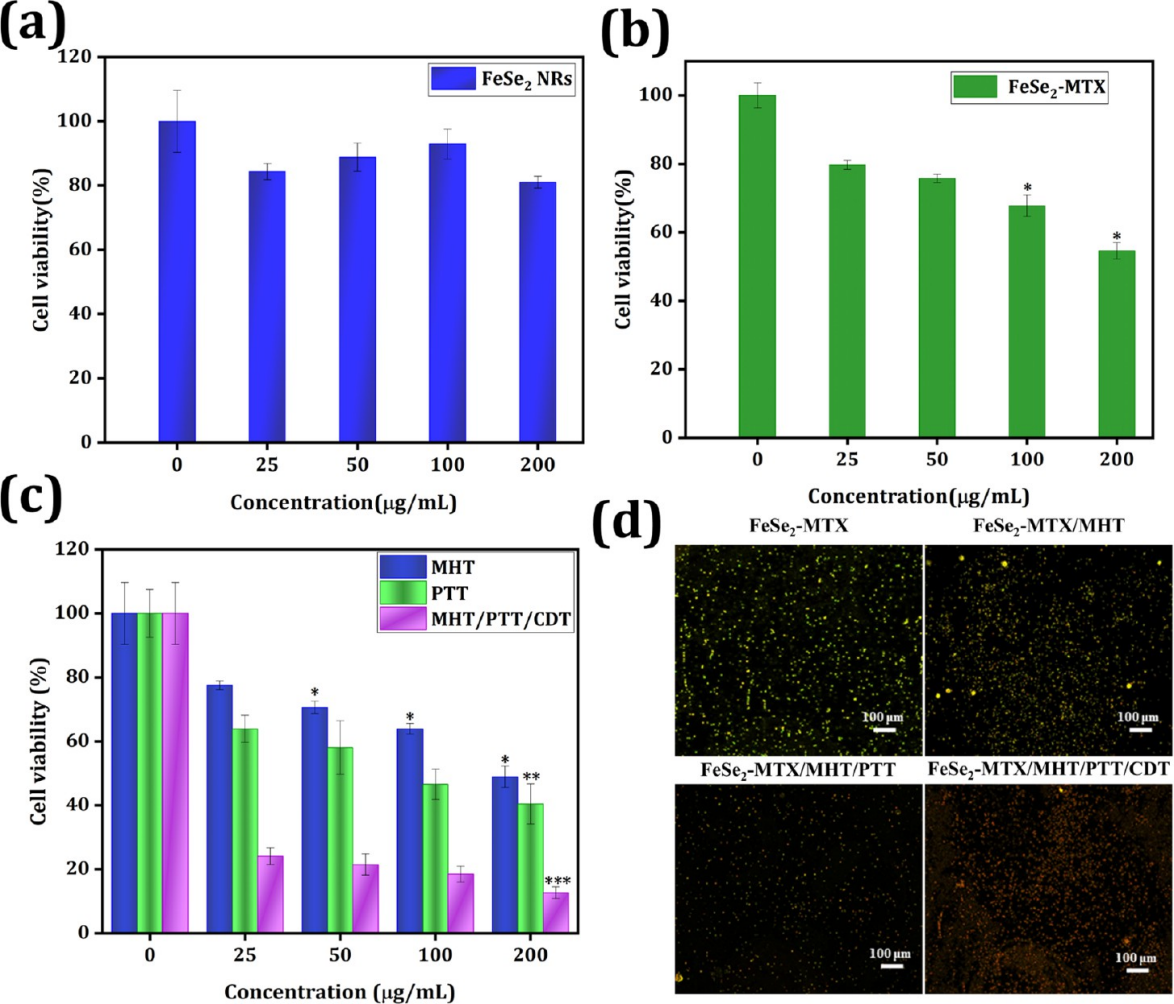
In vitro cell experiments. (a, b) Cell viability of MCF-7 cells incubated with FeSe_2_ and FeSe_2_-MTX for 24 h. (c) Impact of synergistic MHT/PTT/CDT on MCF-7 cells with FeSe_2_-MTX. The labeled asterisk represents statistical significance compared with the control via one-way analysis of variance (ANOVA) with the Tukey post hoc test. *p < 0.05, **p < 0.01, ***p < 0.001. (d) Cells after live/dead staining observed via FM.

The biosafety of nanomaterials is a key parameter in biomedical applications. To further examine the in vitro cytotoxicity of FeSe_2_ and FeSe_2_-MTX, MCF-7 cells were treated with various concentrations of FeSe_2_ and FeSe_2_-MTX for 24 h, and the MTT assay was used to measure the cytotoxicity. FeSe_2_ NRs were biocompatible, as there was no apparent cytotoxicity against L929 cells in the range of 0–200 μg/mL (Figure S7). Different cell lines may exhibit varying sensitivities to reactive oxygen species (ROS) and oxidative stress. L929 fibroblast cells may have different intracellular environments and antioxidant defense mechanisms from cancer cells, such as MCF-7. The Fenton reaction requires transition-metal ions, particularly iron. Differences in iron metabolism between normal and cancer cells may contribute to the variations in their susceptibility to Fenton-like reactions. Normal cells regulate iron efficiently, thereby preventing excessive ROS generation. Normal cells often possess robust antioxidant defense mechanisms that neutralize ROS and prevent oxidative damage. These defense mechanisms may be more effective in L929 cells, thus mitigating the potential cytotoxic effects of FeSe_2_ NRs. The Fenton reaction is pH-dependent, and the intracellular pH of L929 cells may be within a range that is not conducive to efficient Fenton chemistry. This reaction typically occurs more readily under acidic conditions. The redox status of cells, which reflects the balance between oxidants and antioxidants, differs among the cell types. L929 cells may maintain a more balanced redox state, making them less susceptible to the oxidative stress induced by FeSe_2_ NRs. In summary, the lack of apparent cytotoxicity in L929 cells in response to FeSe_2_ NRs may be attributed to a combination of factors related to the specific characteristics and regulatory mechanisms of normal fibroblasts. Understanding these factors is crucial to interpreting the selectivity and efficacy of FeSe_2_ NRs as potential Fenton agents for cancer treatment. However, when MCF-7 cells were treated with 200 μg/mL FeSe_2_ NRs, the cell survival rate dropped to 80%, suggesting that FeSe_2_ NRs, as a Fenton agent, may catalyze the generation of ^•^OH in cancer cells, thereby destroying cancer cells, as shown in Figure 6a. At lower concentrations, the cellular uptake of FeSe_2_ NRs may not have reached saturation, resulting in a gradual increase in cytotoxicity with increasing concentration. However, at higher concentrations, cellular uptake may become saturated, leading to an increase in cytotoxic effects. The dynamics of intracellular ROS generation and accumulation can be complex. At lower concentrations, FeSe_2_ NRs may induce a moderate increase in ROS, whereas at higher concentrations, ROS levels may reach elevated levels owing to factors such as antioxidant defenses or cellular adaptations.

As shown in Figure 6b, the percentage of viable MCF-7 cells in FeSe_2_-MTX at a concentration of 200 μg/mL was 45.1%, confirming the enhanced anticancer effects of the NRs upon MTX modification. This is because chemotherapeutic drugs are released from nanocarriers and diffuse into the nucleus from the cytosol, thereby interfering with DNA replication and causing cell death.^11^ The therapeutic efficacy of FeSe_2_-MTX in MCF-7 cells was later examined. At a concentration of 200 μg/mL, the combination of MHT/PTT/CDT exhibited a remarkable synergistic inhibitory effect by substantially reducing the viability of cancerous cells to 19%. Although a single therapy could also exert an anticancer effect, its anticancer efficacy was much lower than that of the MHT/PTT/CDT combination (Figure 6c), possibly because of the photothermally enhanced chemotherapy and the Fenton/Fenton-like reaction catalytic efficiency.

A double-staining approach was used to verify the cytotoxic effects of the combined MHT/PTT/CDT treatment by fluorescently labeling living and dead cells with green and red dyes, respectively. The group treated with the MHT/PTT/CDT combination had no viable cells. However, the majority of cancer cells were killed following a single treatment, compared to the groups treated with FeSe_2_-MTX alone (Figure 6d). ROS images obtained by 2′-7′-dichlorodihydrofluorescein diacetate (DCFH-DA) staining (Figure S8) of MCF-7 cells after treatment to confirm the generation of hydroxyl radicals by showing green fluorescence are presented in the supporting document (Figure S8). Hydroxyl radicals (^•^OH) are highly reactive and damage the ROS. These metal ions participate in the Fenton reaction, generating hydroxyl radicals by reacting with hydrogen peroxide (H_2_O_2_)
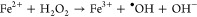
DCFH-DA can react with ROS, including hydroxyl radicals, to form the highly fluorescent compound 2′,7′-dichlorofluorescein (DCF). This reaction leads to a significant increase in fluorescence, which can be detected and quantified using fluorescence microscopy. These results indicated that FeSe_2_-MTX possesses excellent anticancer activity in vitro because of its synergistic MHT/PTT/CDT action.

### 3.6 MRI In Vitro and In Vivo

**Figure 7:**
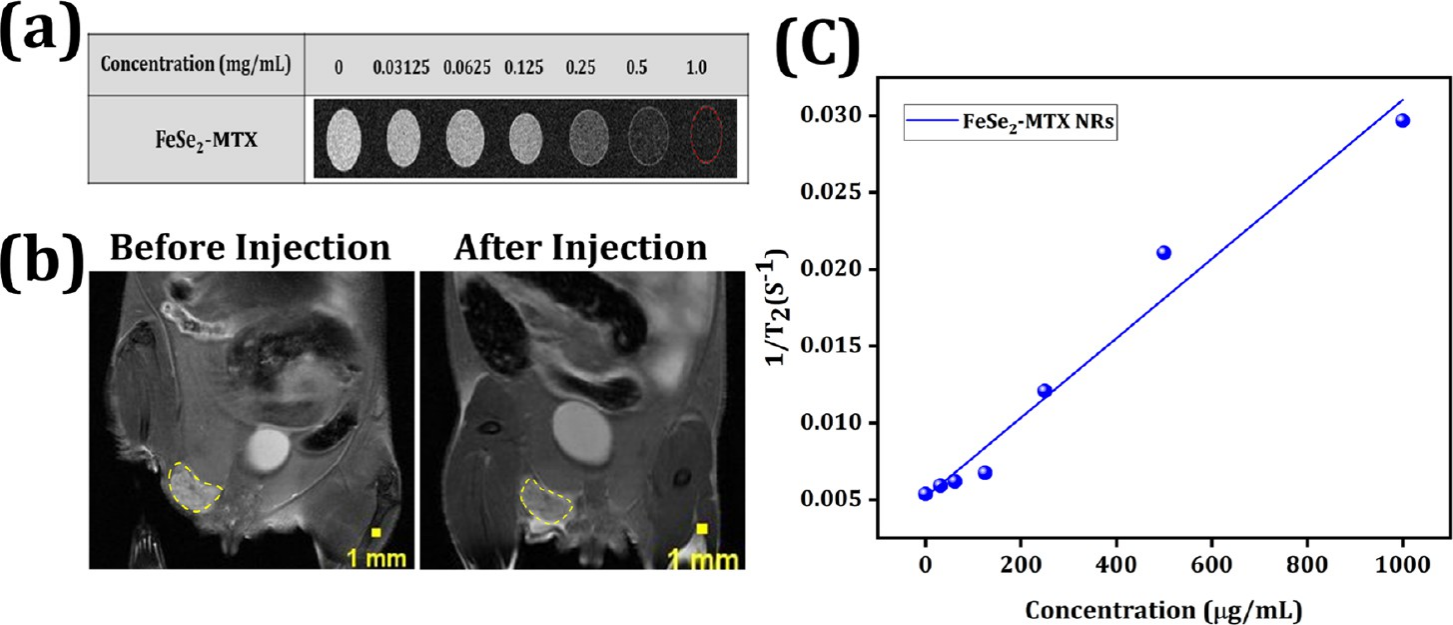
MRI. (a) In vitro *T*_2_-weighted MRI images of FeSe_2_-MTX at different concentrations. (b) In vivo *T*_2_-weighted MRI before and after intravenous injection. (c) Transverse relaxivity value of FeSe_2_-MTX.

MNPs (paramagnetic or superparamagnetic) are often used as contrast agents for in vivo MRI. MRI provides extensive information on tumors for pretreatment assessment, enabling real-time observation of therapeutic progress, evaluation of therapeutic effects, and appropriate cancer treatment. First, the MRI capability of FeSe_2_ NRs was examined. The in vitro MRI phantoms progressively became hypotensive as the Fe concentration increased (Figure 7a). We also measured the *T*_2_-weighted signal intensity of FeSe_2_ NRs with various Fe concentrations and found a linear relationship between the 1/*T*_2_ intensity and Fe concentration, with a relaxivity *r*_2_ of 2.58 mg^–1^ s^–1^ (Figure 7c). This *r*_2_ value suggests that the nanosystem can enhance the contrast in *T*_2_-weighted MRI images.

Next, we assessed its potential application in the in vivo MRI of a xenograft tumor model (Figure 7b). *T*_2_-weighted MRI images of the tumor regions were acquired before and after injection of the NRs. Two hours after the intravenous injection of FeSe_2_-MTX, the tumor became darker than before the injection. These results suggest the potential utility of FeSe_2_-MTX for tumor-targeting MRI in vivo.

### 3.7 In Vivo Therapeutic Efficacy

**Figure 8:**
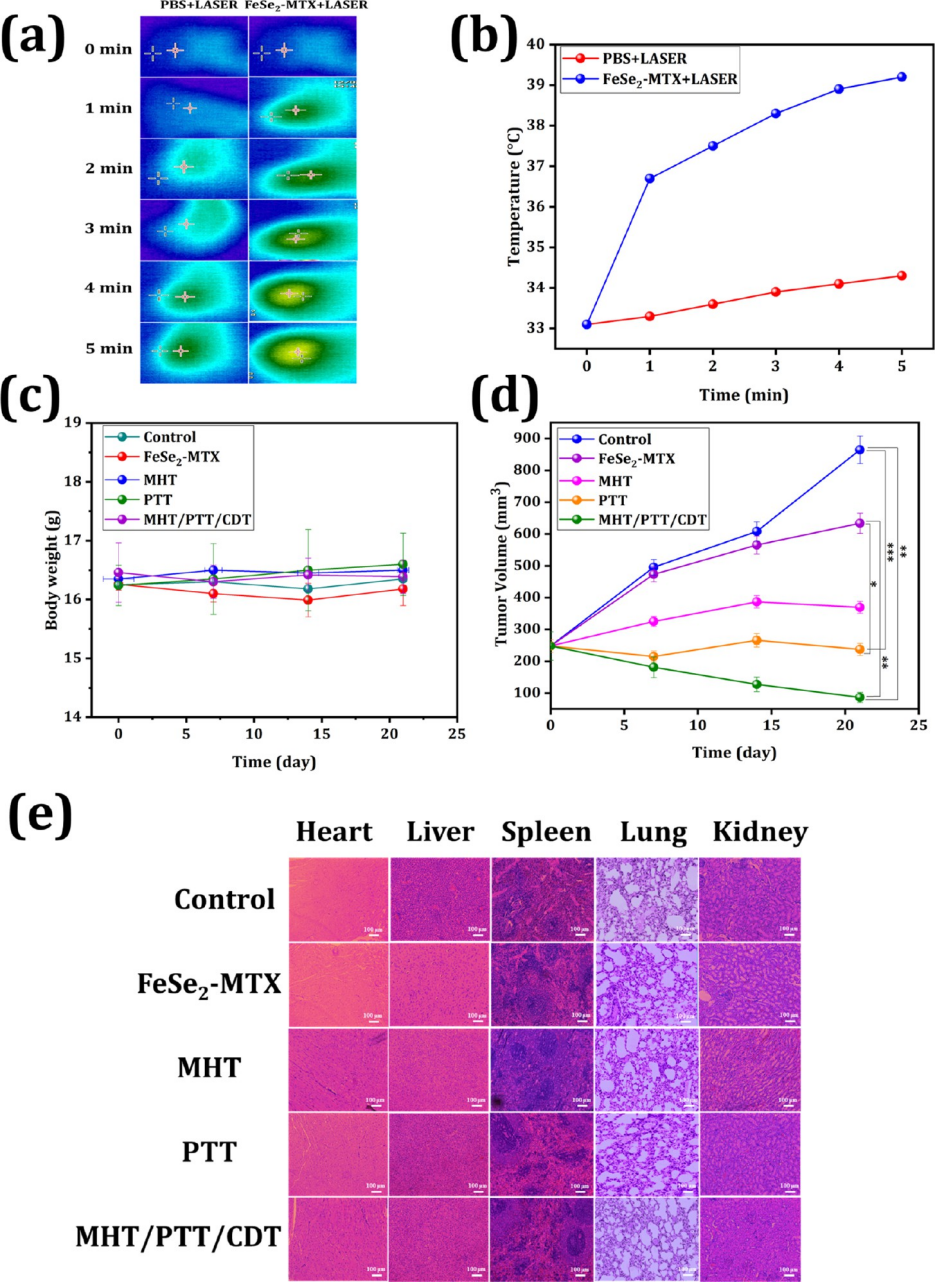
In vivo therapeutic efficacy. (a, b) Thermal images and temperature elevation in tumor-bearing mice under NIR irradiation. (c) Body weight analysis of mice in different treatment groups. (d) Tumor volume of mice in different treatment groups. The labeled asterisk represents statistical significance compared with the control via one-way ANOVA with the Tukey post hoc test. *p < 0.05, **p < 0.01, ***p < 0.001. (e) H&E staining of major organs.

Using MCF-7 cancer xenografts, we examined the in vivo anticancer efficacy of the synergistic MHT/PTT/CDT. First, the in vivo photothermal therapeutic efficacy of FeSe_2_-MTX was studied. Nude mice with MCF-7 tumors were randomly assigned to two groups: PBS and FeSe_2_-MTX + laser treatment. Each group was subjected to laser irradiation for 5 min, and thermal images and temperatures were captured using an IR camera. As depicted in Figure 8a,b, mice in the FeSe_2_-MTX + laser group showed a dramatic increase in tumor site temperature after 5 min of irradiation. The temperature in the FeSe_2_-MTX + laser group increased slowly, reaching 39.2 °C in 5 min, which was sufficient to ablate the neoplasm. In contrast, only a minor increase in temperature was observed in the PBS group. These results indicate that FeSe_2_-MTX is beneficial for in vivo photothermal therapy.

Biosafety is a requirement for the safe application of materials in the biomedical field, which led us to examine the in vivo toxicity of FeSe_2_-MTX. High toxicity typically leads to weight loss. The body weights of the mice in each treatment group were measured. Figure 8c shows negligible variation in the average weights between the groups, confirming the biocompatibility of our materials in vivo. In addition, the therapeutic efficacy of the MHT/PTT/CDT combination was evaluated in vivo by observing changes in the tumor volume after 21 days of treatment. Mice treated with FeSe_2_-MTX and laser irradiation (808 nm) exhibited only mild tumor suppression compared to the control groups (Figure 8d). In contrast, MHT/PTT/CDT combination therapy significantly inhibited tumor growth. After 21 days of treatment, the major organs in the different treatment groups were collected and examined (Figure 8e). Biodistribution analysis of the nanoparticles at 24 h and the corresponding results are displayed in the Supporting Information (Figure S9). MCF-7 tumor-bearing mice were intravenously injected with FeSe_2_-MTX. The residual Fe in the main organs and tumor after 24 h was determined by inductively coupled plasma mass spectrometry (ICP-MS). The data demonstrate a substantial decrease in the accumulation of FeSe_2_-MTX in the tissues, as illustrated in Figure S9. This suggests that the material may undergo continuous degradation and clearance in mice.^62^ Digital images of the tumors are shown in Figure S10.

No apparent pathological differences were observed between the control and the treated groups, indicating the safety of the technique. Overall, these results demonstrate the efficacy of MHT/PTT/CDT combination therapy for tumor suppression. H&E and terminal deoxynucleotidyl transferase dUTP nick end labeling (TUNEL) (Figure S11) were used to observe the pathological changes in the tumor. In the control group, no significant damage to the tumor tissues was observed. However, in the MHT/PTT/CDT group, there was a clear impairment of tumor tissue integrity. Notably, TUNEL staining revealed severe destruction, including nuclear damage, indicating a high rate of cell necrosis and apoptosis in tumors treated with the combined MHT/PTT/CDT therapy. This effect was attributed to the generation of ROS through a Fenton-like reaction and the synergistic MHT/PTT treatment. These results suggested a synergistic therapeutic effect of the nanorods. These results confirmed that FeSe_2_-MTX is a biocompatible material that is safe for in vivo cancer diagnosis and treatment.

The limitation of the manuscript is unfortunately, due to the pandemic situation and resource limitations, we and neighboring universities were unable to perform these additional analyses to provide a more comprehensive understanding of the biological effects of FeSe_2_-MTX NRs. The evaluation of blood parameters, such as red blood cells, white blood cells, platelets, liver enzymes (ALT, AST), and kidney function markers (BUN, creatinine), would have offered valuable insights into the potential toxicity of the NRs. Without this data, our assessment of the biosafety profile of these nanoparticles is limited. Future studies should aim to incorporate a more extensive panel of biomarkers, including inflammatory cytokines, oxidative stress indicators, and histological examinations of major organs. This would enable a thorough investigation of the potential systemic effects and safety of FeSe_2_-MTX NRs, which is crucial for their further development and clinical translation.

## 4 Conclusions

In conclusion, our study demonstrated the applicability of FeSe_2_-MTX in the treatment of breast cancer MHT/PTT/CDT. The FeSe_2_ NRs were remotely activated using a NIR laser to achieve an effective heat conversion. To improve the efficacy of drug delivery to cancer cells, FeSe_2_ NRs were loaded with the anticancer drug MTX, which exhibited a high loading capacity owing to their physical interactions. Furthermore, FeSe_2_ NRs acted as good contrast agents for the MRI of tumors in vivo. Notably, simultaneous MHT/PTT/CDT caused cell death in vitro and total tumor ablation in vivo under a magnetic field at an optimum laser power. The PTT performance further increased the efficacy of CDT. Despite this limitation, the results obtained offer valuable contributions to the field, demonstrating potential implications and avenues for future research. Therefore, heat therapy combined with CDT may be an effective method for treating tumors with minimal collateral damage. Therefore, the unique nanoplatform developed in this study can be used for the accurate diagnosis and effective treatment of breast cancer.

## Supporting Information Available

Materials and methods (experimental), DLS analysis, ζ potential analysis, FTIR spectra of FeSe_2_, pure MTX, FeSe_2_-MTX, UV–vis spectra of FeSe_2_-MTX and free MTX, high-resolution O 1s spectra of FeSe_2_ NRs, drug release from FeSe_2_-MTX at pH 6.4, biocompatibility analysis of FeSe_2_ NRs on L929 cells, ROS analysis of MCF-7 cells with different treatments, biodistribution analysis of FeSe_2_-MTX, digital images of tumors after different treatments, and H&E and TUNEL staining of tumors with different treatments (PDF)
