# Supplementary material for: NIR-Responsive Methotrexate-Modified Iron Selenide Nanorods for Synergistic Magnetic Hyperthermic, Photothermal, and Chemodynamic Therapy
Source: ACS Appl Mater Interfaces. 2024 May 13;16(20):25622–36. doi: 10.1021/acsami.3c18450 (PMC11129116; doi:10.1021/acsami.3c18450)
Supplement: Supplementary file 1 — am3c18450_si_001.pdf [file am3c18450_si_001.pdf]

Supporting Information

NIR-Responsive Methotrexate-Modified Iron Selenide Nanorods for Synergistic Magnetic Hyperthermic, Photothermal, and Chemodynamic Therapy

Senthilkumar Thirumurugan [1,†], Kayalvizhi Samuvel Muthiah [1,†], Yu-Chien Lin [1], Udesh Dhawan [2], Wai-Ching Liu [3], An-Ni Wang [4], Xinke Liu [5,6], Michael Hsiao [7,8], Ching-Li Tseng [9,10,11,12], Ren-Jei Chung [1,13,*]

[1] Department of Chemical Engineering and Biotechnology, National Taipei University of Technology (Taipei Tech), Taipei 10608, Taiwan

[2] Centre for the Cellular Microenvironment, Division of Biomedical Engineering, James Watt School of Engineering, Mazumdar-Shaw Advanced Research Centre, University of Glasgow, Glasgow G116EW, UK

[3] Faculty of Science and Technology, Technological and Higher Education Institute of Hong Kong, New Territories, Hong Kong 999077, China

[4] Scrona AG, Grubenstrasse 9, 8045 Zürich, Switzerland

[5] College of Materials Science and Engineering, Chinese Engineering and Research Institute of Microelectronics, Shenzhen University, Shenzhen 518060, China

[6] Department of Electrical and Computer Engineering, National University of Singapore, Singapore 117583, Singapore

[7] Genomics Research Center, Academia Sinica, Taipei 115, Taiwan

[8] Department and Graduate Institute of Veterinary Medicine, School of Veterinary Medicine, National Taiwan University, Taipei 10617, Taiwan

[9] Graduate Institute of Biomedical Materials and Tissue Engineering, College of Biomedical Engineering, Taipei Medical University, Taipei 11031, Taiwan.

[10] International Ph. D. Program in Biomedical Engineering, College of Biomedical Engineering, Taipei Medical University, Taipei 11031, Taiwan.

[11] Research Center of Biomedical Device, College of Biomedical Engineering, Taipei Medical University, Taipei 11031, Taiwan.

[12] International Ph. D. Program in Cell Therapy and Regenerative Medicine, College of Medicine, Taipei Medical University, Taipei 11031, Taiwan.

[13] High-value Biomaterials Research and Commercialization Center, National Taipei University of Technology (Taipei Tech), Taipei 10608, Taiwan

† These authors contributed equally to this paper.

* Corresponding Author:
Prof. Ren-Jei Chung
Email: rjchung@mail.ntut.edu.tw; Tel: (886-2) 2771-2171 ext. 2547
Address: Department of Chemical Engineering and Biotechnology, National Taipei University of Technology (Taipei Tech), No. 1, Sec. 3, Zhongxiao E. Rd., Taipei 10608 Taiwan.

Materials and methods

Chemicals and reagents

[Fe $(NO_3)]_3.9H_2O$ (75 mM), Na_2SeO_3 (75 mM), and hydrazine hydrate ($N_2H_4.H_2O$, 1.5 M) were all purchased from Sigma Aldrich (USA). Hydrogen peroxide (30% H2O2), Methylene Blue (MB), 5,5-Dimethyl-1-pyrroline N-oxide (DMPO) and MTT (3-(4, 5-dimethylthiazolyl-2)-2, 5-diphenyltetrazolium bromide) were purchased from Sigma Aldrich (USA).

Characterization of $FeSe_2$ NRs

Transmission electron microscopy (TEM) and Field emission scanning electron microscope (FE-SEM) was used to characterize the size and structural morphology of $FeSe_2$ NRs. EDX analysis was used to determine the presence of elements in as-prepared NPs. Additionally, X-ray diffraction (XRD) and X-ray photoelectron spectroscopy (XPS) were used to establish the crystalline nature and oxidation states of $FeSe_2$ NRs. The presence of functional groups was determined using Fourier transform-infrared spectroscopy (FTIR). Finally, SQUID was used to analyze the magnetic characteristics of NRs. Using Raman spectroscopy (Ramboss 500i Micro (DINGWOO/USA)), the chemical compositions of as-prepared materials were determined.

$FeSe_2$ NRs with Amine Functionalization

To summarize, 200 mg $FeSe_2$ NRs were dissolved in 100 mL ethanol. Then, 1ml of APTES was added to the mixture mentioned above, and the mixture was sonicated at 60°C for 4 hours. Following cooling, the combinations were washed twice with ethanol and water and dried in vaccum.

Modification of drugs into NRs

50mg of amine-functionalized NRs were mixed with 20ml of DMSO solution (1mg/mL) to load the anticancer drug MTX. Following that, 17ml of EDC/NHS was added to the combination described above, then the solution's pH was adjusted to 8.2. Further, the solution was incubated overnight at 37°C in the dark. Subsequently, FTIR analysis was used to establish that MTX was functionalized into NRs.

Detection of Hydroxyl Radical (·OH) Generation

The ·OH signal was detected using an ESR spectrometer with 5,5-Dimethyl-1-pyrroline N-oxide (DMPO) as the·OH trapping agent. The experimental groups were H_2O_2, H_2O_2+ $FeSe_2$ NRs (pH 7.4), and H_2O_2+ $FeSe_2$ NRs (pH 6.5), respectively. The final concentrations of $FeSe_2$ NRs and H_2O_2 were 100 μg mL^{-1} and 2 mM, respectively.

·OH *generation through $FeSe_2$-MTX based fenton-like reaction*

0.2 mg of $FeSe_2$-MTX was added to 10 µL H_2O_2 (1 M) and 1 mL MB solution (10 µM) at a pH of 6.5. After incubation for 20 minutes at room temperature, the mixed solution was centrifuged to remove $FeSe_2$-MTX, and UV–vis spectroscopy was used to measure the absorbance at 661 nm.

PTT conversion efficiency

To determine the photothermal conversion efficiency (η), the difference in temperature versus time was monitored under laser irradiation (808 nm) at 2 W/cm^2 until the solution reached a stable state. After closing the irradiation source, the rate at which heat was transferred from the solution to the surrounding environment was determined by measuring the drop in temperature of the aqueous solution using the following equation:[62]

$$\eta = \frac{hS\,(Tmax - Tsurr) - Qdis}{I(1 - 10^{-A_{808}})} \longrightarrow (1)$$

where h and S indicate the heat transfer coefficient and surface area of the container, respectively, Tmax is the maximum temperature, and Tsurr is the temperature of the environment. Q_{dis} is the heat accompanying the absorbance of light, which was individually calculated using a cuvette cell comprising purified water in the absence of the prepared materials, I is the power density of the laser (2 W/cm^2), and A_{808} is the absorbance of $FeSe_2$ at 808 nm (A_{808} = 0.2482). The hS value was calculated using the following equation:[62]

$$\tau s = \frac{mD\ CD}{hS} \longrightarrow (2)$$

where Ꞇs is the constant time of the sample, mD and CD indicate the mass and heat capacity of purified water, respectively.

Cellular uptake of NRs

Preparation of fluorescently labeled $FeSe_2$- MTX

Nanocarriers were fluorescently labeled with fluorescein isothiocyanate (FITC) by progressively adding a methanolic solution of FITC to the appropriate concentrations of $FeSe_2$- MTX in PBS (pH 7.4) at ambient temperature for 24 h in the dark. Then, FITC-conjugated $FeSe_2$ -MTX was washed and dried.

Fluorescence microscopy (FM) analysis

Confluent MCF-7 cells were grown on coverslips in 35-mm culture dishes at a concentration of 4 × 10^5 cells. Cells were then treated with the $FeSe_2$-MTX/FITC conjugate at different doses (25, 50, 100, and 200 µg mL^{-1}) under the same conditions.

After 4 h, cells were washed and fixed with 4% formaldehyde 5 min at room temperature. Then, 4´-6-diamidino-2-phenylindole (DAPI) was used to visualize the nuclei of cells because it entered the DNA and acted as a nuclear marker. Cells were then washed and stained with the DAPI/PBS solution for 5 min. Finally, FM was performed to study the cells. Excitation and emission wavelengths of DAPI were 364 and 461 nm, respectively, and those of FITC were 488 and 518 nm, respectively.

Cell culture

In a T75 flask, Human Breast Cancer (MCF-7) cells (ATCC, USA) were grown in a DMEM medium accompanied with 10% FBS (GIBCO, USA), 100 µg/mL penicillin, and 100 µg/mL of streptomycin. The incubators were maintained at a temperature of 37°C with 5% CO_2 having a relative humidity of 95%.

Cell Viability

The $FeSe_2$ NRs, $FeSe_2$ – MTX were subjected to an in vitro cytotoxicity experiment using MTT. MCF-7 cells have been plated at a concentration of 5×10^4 cells/well in a 96-well plate. After 12 hours of culturing cells, the media has been removed, and the $FeSe_2$, $FeSe_2$ – MTX NRs in DMEM were reintroduced to the 96-well plate. The final concentrations were 0, 25, 50, 100, and 200 µg mL^{-1}, respectively, with a final medium volume of 100 µL in each well. After 24 hours of incubation, 100 µL of MTT solution were added to each well. Following that, the absorbance at 570 nm was determined using a microplate reader. Cell viability was quantified as the difference in the percentage of viable cells between treated and untreated control cells.

Statistical analysis

Experiments were conducted thrice independently using the nanoparticles. The data is represented as a mean with standard deviations. The statistical data results were

analyzed using Statistical Package for Social Sciences (SPSS 18.0 for windows, SPSS Inc., Chicago, IL, USA); one-way ANOVA was utilized to identify statistically different datasets. Significant data sets with p-value <0.05 are represented with * and those with p-value <0.01, $<0.001$and <0.0001 were represented with **, *** or ***, respectively.

Supporting Figures:

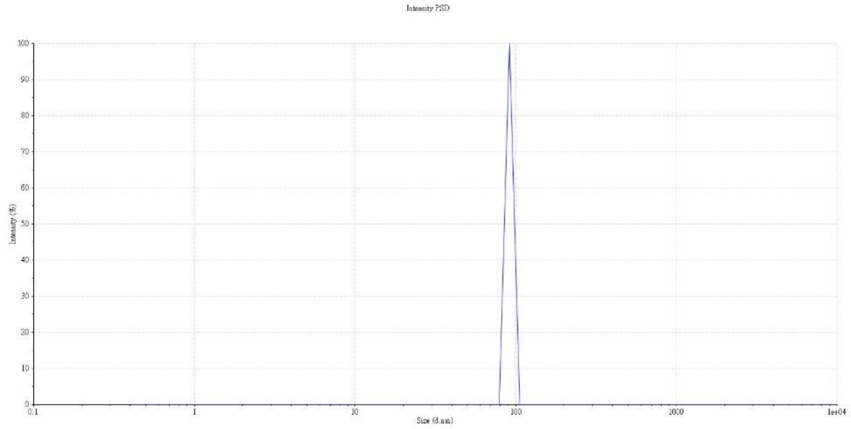

Figure S1. DLS analysis.

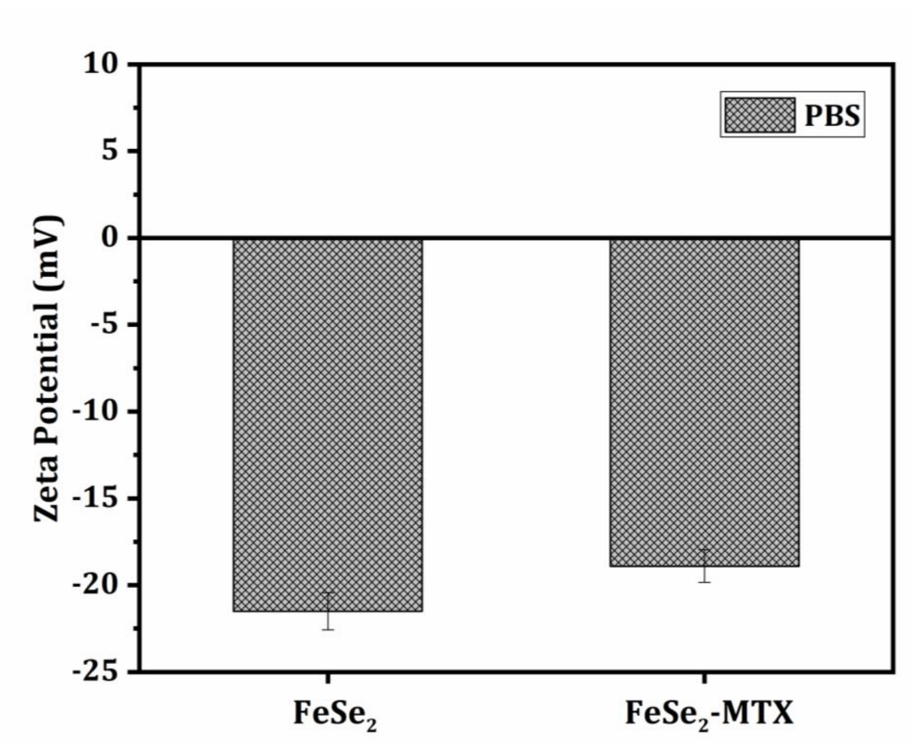

Figure S2. (a) Zeta potential analysis of as-prepared materials.

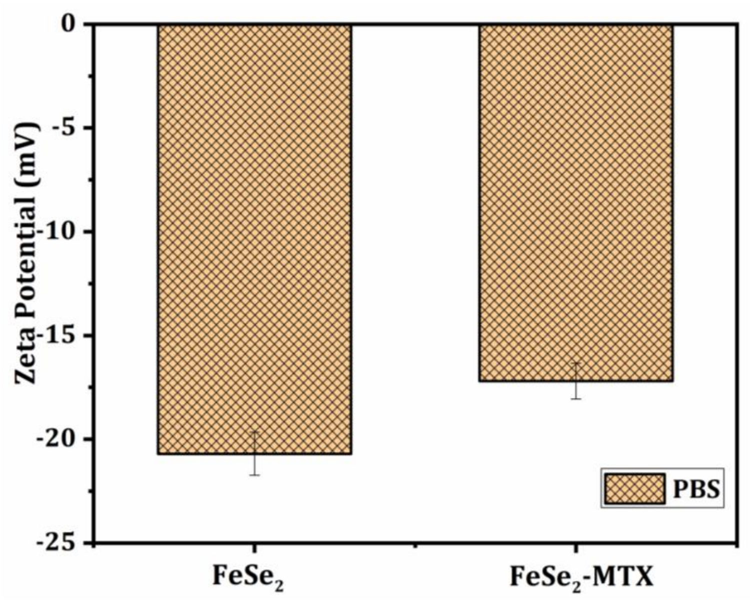

Figure S2. (b) Zeta potential analysis of materials after one week storage.

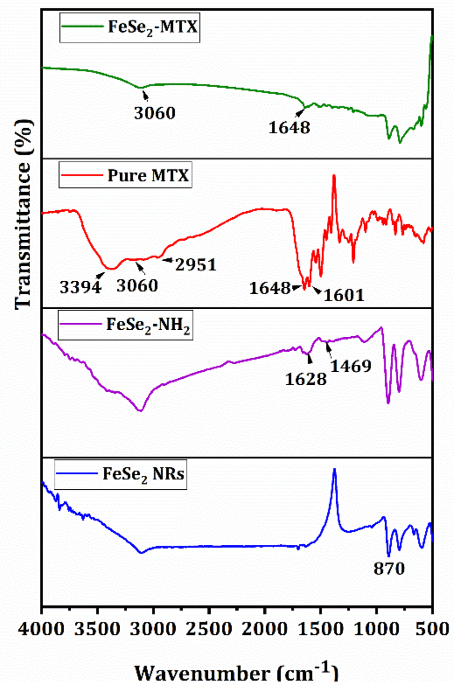

Figure S3. FTIR spectra of $FeSe_2$, pure MTX, $FeSe_2$-MTX.

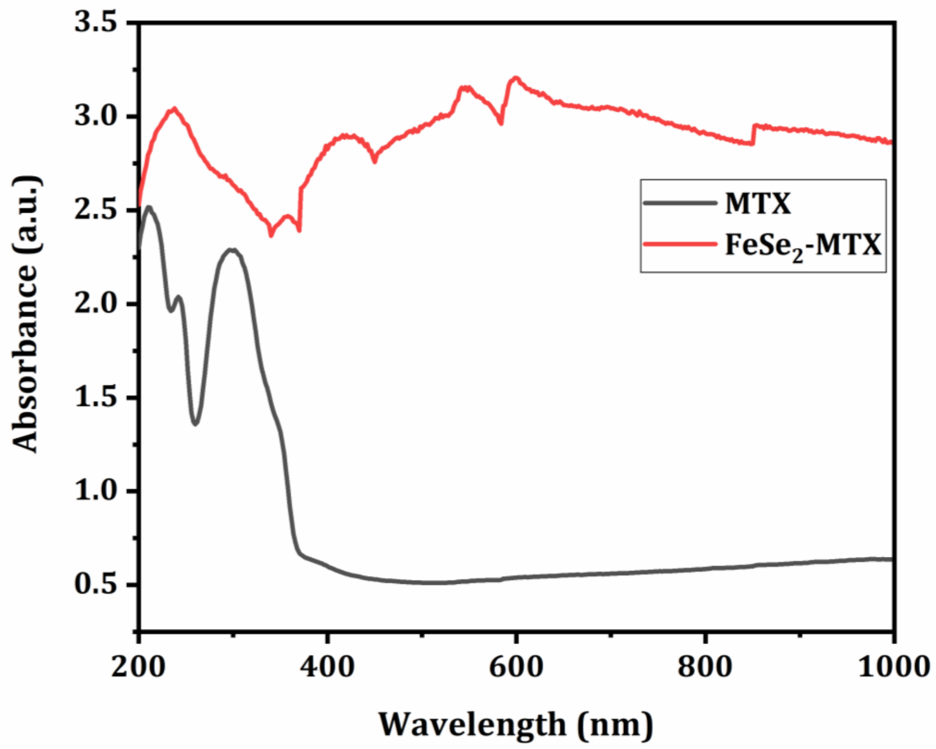

Figure S4. UV-Vis spectra of $FeSe_2$-MTX and free MTX.

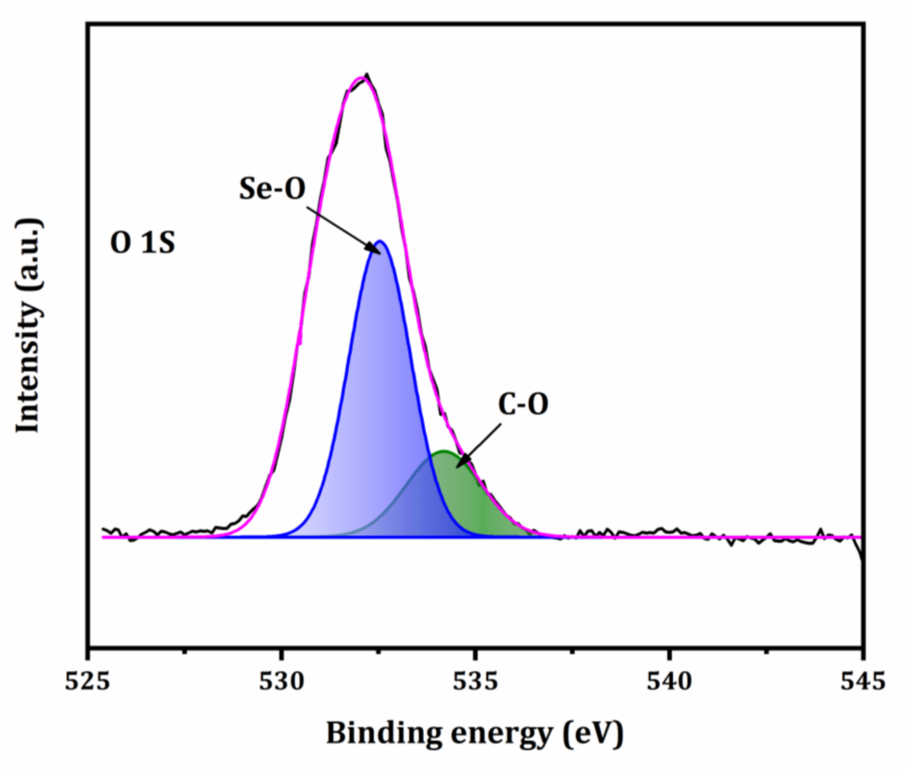

Figure S5. High resolution O 1s spectra of $FeSe_2$ NRs.

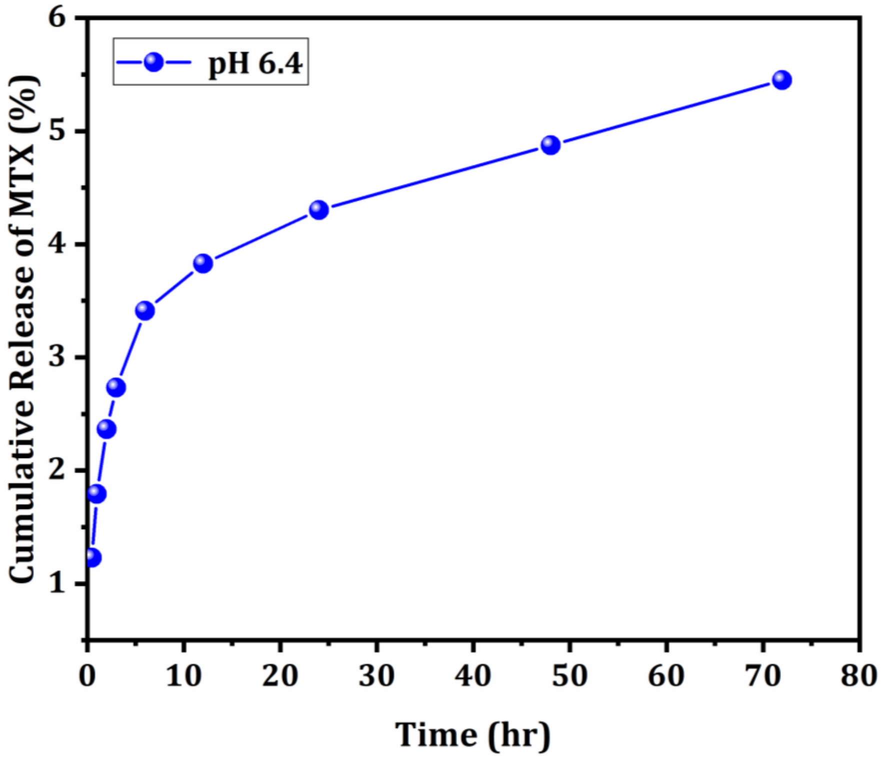

Figure S6. Drug release from FeSe2-MTX at pH 6.4.

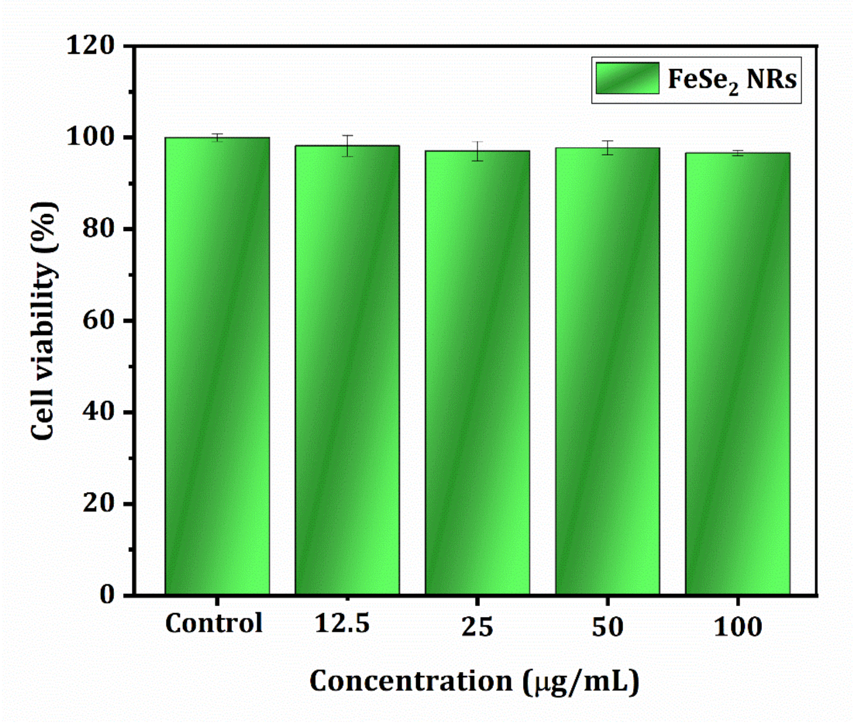

Figure S7. Biocompatibility analysis of $FeSe_2$ NRs on L929 cells.

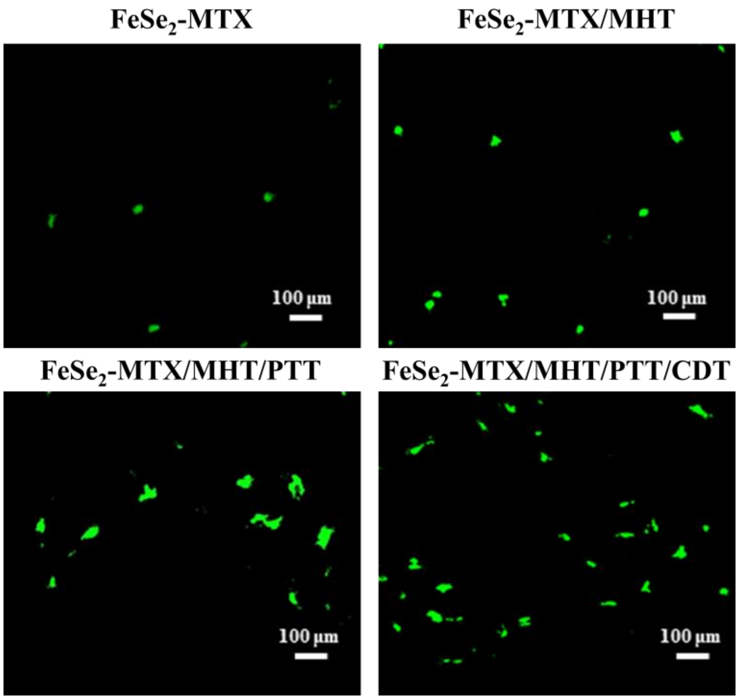

Figure S8. ROS analysis of MCF-7 cells with different treatments.

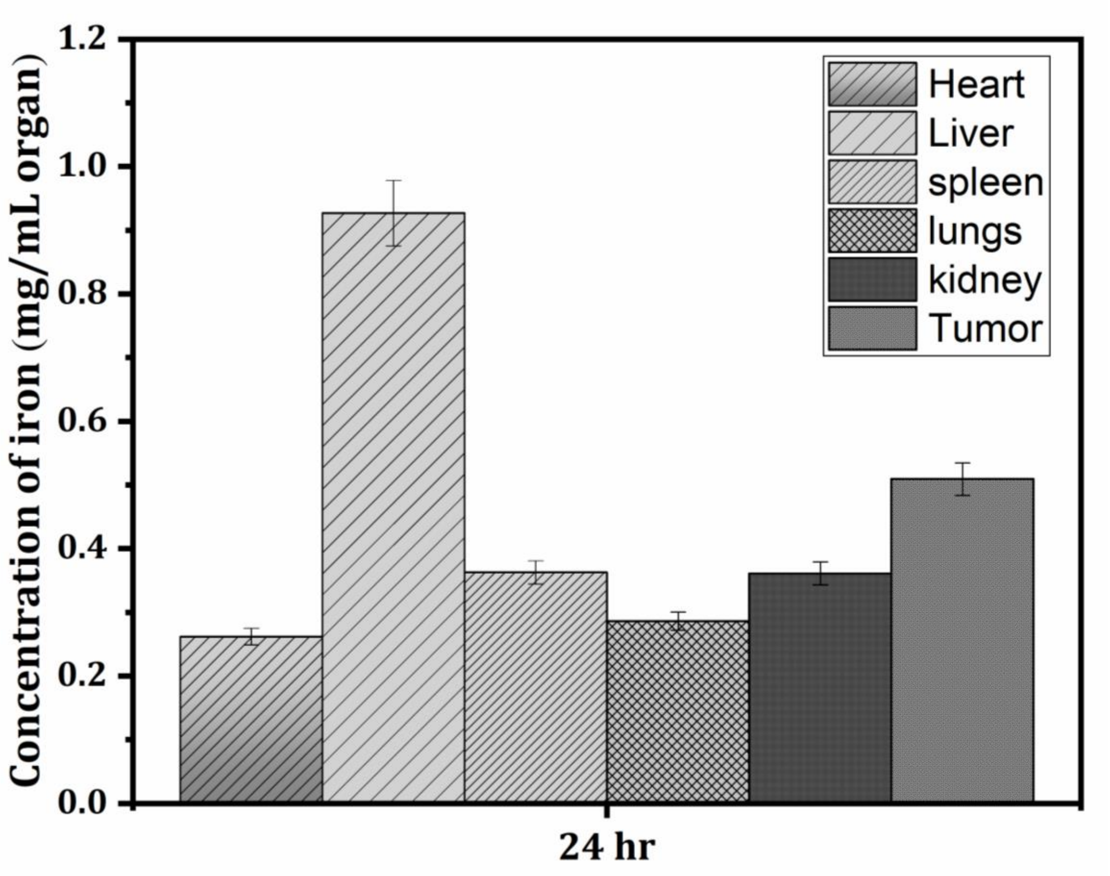

Figure S9. Biodistribution analysis of $FeSe_2$-MTX.

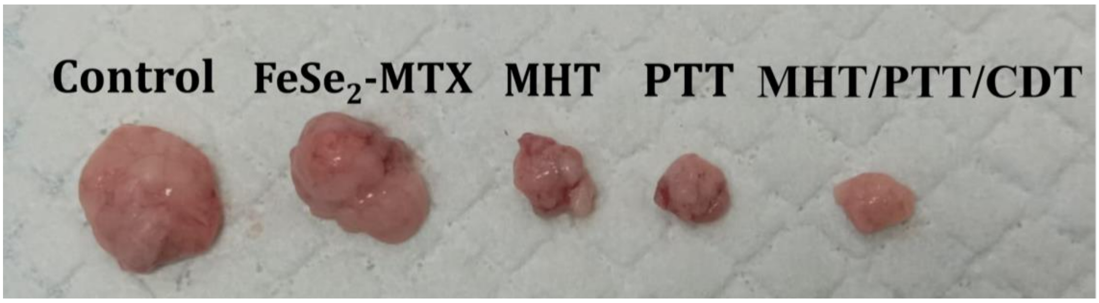

Figure S10. Digital images of tumors after different treatments.

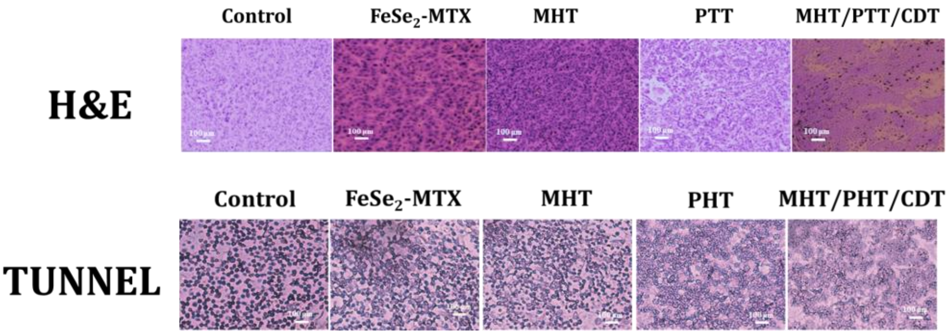

Figure S11. H&E and TUNNEL staining of tumors with different treatments.
